# Smartphone-based symbol-digit modalities test reliably captures brain damage in multiple sclerosis

**DOI:** 10.1038/s41746-021-00401-y

**Published:** 2021-02-24

**Authors:** Linh Pham, Thomas Harris, Mihael Varosanec, Vanessa Morgan, Peter Kosa, Bibiana Bielekova

**Affiliations:** Neuroimmunological Diseases Section (NDS), Laboratory of Clinical Immunology and Microbiology (LCIM), National Institute of Allergy and Infectious Diseases (NIAID), National Institutes of Health (NIH), Bethesda, MD USA

**Keywords:** Multiple sclerosis, Multiple sclerosis

## Abstract

As the burden of neurodegenerative diseases increases, time-limited clinic encounters do not allow quantification of complex neurological functions. Patient-collected digital biomarkers may remedy this, if they provide reliable information. However, psychometric properties of digital tools remain largely un-assessed. We developed a smartphone adaptation of the cognitive test, the Symbol-Digit Modalities Test (SDMT) by randomizing the test’s symbol-number codes and testing sequences. The smartphone SDMT showed comparable psychometric properties in 154 multiple sclerosis (MS) patients and 39 healthy volunteers (HV). E.g., smartphone SDMT achieved slightly higher correlations with cognitive subscores of neurological examinations and with brain injury measured by MRI (*R*^2^ = 0.75, Rho = 0.83, *p* < 0.0001) than traditional SDMT. Mathematical adjustment for motoric disability of the dominant hand, measured by another smartphone test, compensates for the disadvantage of touch-based test. Averaging granular home measurements of the digital biomarker also increases accuracy of identifying true neurological decline.

## Introduction

With an aging population, the prevalence of chronic neurological diseases is projected to increase dramatically. Most countries already experience a shortage of neurologists. By 2025 in the United States, the average demand for neurologists is expected to eclipse the supply by 20% or more^1^.

Current solutions of shifting care for neurological patients to primary care providers, while simultaneously decreasing the length of individual patient-encounters for neurologists, lead to delays or mistakes in diagnoses and suboptimal patient outcomes. Indeed, a comprehensive neurological examination takes 40–60 min to perform and years to master. Consequently, such a demanding exam is rarely performed in contemporary clinical practice, depriving patients of reliable measurements of their disease progression necessary for optimal therapeutic decisions.

An alternative solution is to develop a surrogate of neurological examination which is accessible, reliable, and sensitive to changes in disease course. To meet this need, we have created a collection of smartphone tests, the Neurological Functional Test Suite (NeuFun), administered in a patient-autonomous manner^2,3^. NeuFun is designed to recreate the essential domains of neurological examination, allow patients to do testing from home, and have the results forwarded to their clinicians—a capacity essential for telemedicine, e.g., in the times of a global pandemic. Provided that each test in the NeuFun validates its psychometric properties against the gold standard, its use may speed up the identification and referrals of neurological patients from primary care providers to neurologists, help neurologists to focus their examination on affected neurological domains, and reliably track neurological disability during the disease course.

Previous studies have optimized and validated tests that measured motoric and cerebellar functions^2,3^. Here, we have tested psychometric properties of the modified Symbol-Digit Modalities Test (SDMT). The SDMT is a cognitive processing speed test traditionally administered by neuropsychologists^4^. Participants are given a key of nine symbol-digit pairs along with a sequence of symbols. They are then asked to use the key to match as many symbols in the sequence to their corresponding numbers as possible within 90 s. In the written format, participants write the matching numbers on the paper. In the verbal format, participants say the number and test administrators write in participants’ responses. This operator-dependency makes SDMT prohibitively expensive to administer in routine neurology practice. Whereas neuropsychologists administer written followed by oral versions, MS investigators use predominantly oral SDMT to minimize influence of the hand disability on test performance^5^. The common SDMT administration uses a single form, copyrighted by Western Psychological Services, raising a possibility of code memorization on repeated testing, as demonstrated in the oral SDMT for both healthy volunteers (HV) and MS patients^6,7^.

**Fig. 1:**
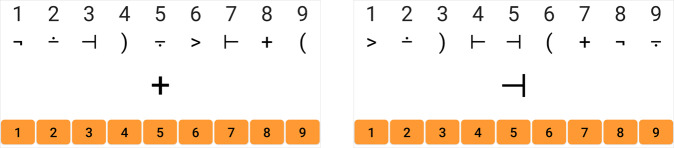
SDMT formatting. The interface for the app based SDMT. The symbol-digit key changes with every trial. During the test, participants only see one symbol at a time and use a virtual number keypad (orange buttons) to input the matching number to the symbol being shown.

To prevent memorization, we introduce several adaptations of the smartphone SDMT. For instance, the symbol-digit key pairing changes randomly with each trial. While this key is displayed on the top of the screen during test duration, participants are shown each symbol (again, randomly generated) one at a time. This prevents inputting the numbers out of sequence in unsupervised testing. To input their responses, participants touch the corresponding number on a virtual number keypad (numpad; Fig. 1). An oral version of the smartphone SDMT uses voice recognition technologies for patients whose motoric disability prevents them from using the numpad.

We also compare the smartphone (and traditional) SDMT to the Paced Auditory Serial Addition Test (PASAT-3), a gold standard that is part of the MS functional composite (MSFC), used in clinical trials. In the PASAT-3, participants are told a sequence of numbers at intervals of 3 s. Participants are asked to add the most recently stated number in the sequence to the prior number and state the result before the next number in the sequence is announced.

## Results

We developed both touch-based and oral versions of smartphone SDMT, and each subject could choose which version they wanted to use. Very few have chosen the oral version of the test, and during the training phase, we noticed that the oral version generated errors when the word-recognition technology failed to correctly identify responses in patients with hypophonia or dysarthria. None of the tested subjects were so disabled that they could not use the touch-based smartphone SDMT. Therefore, we focused on this version and developed mathematical adjustment for motoric dysfunction to compensate for the shortcoming of the touch-based test.

### Smartphone SDMT differentiates HV from MS subjects

**Fig. 2:**
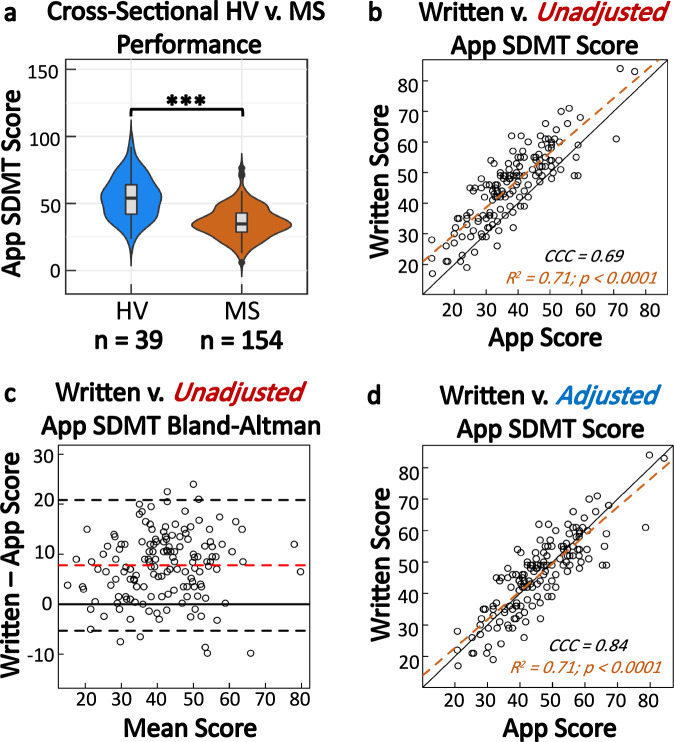
Analysis of app based SDMT validity. **a** HV generally perform better than MS on the app SDMT (*p* < 0.0001). The number of participants for each cohort are shown below the plot. **b** Assessment of agreement between the raw app-based score and the written score (HV = 16; MS = 138). The black solid line is the 1:1 concordance line. The orange dashed line is the regression line. **c** Bland–Altman plot for the written and the app scores. The limits of agreement are −5.29 and 20.84. On average, individuals perform 7.8 points higher on the written test. **d** The plot of concordance between the written and app scores, after adjusting for the 7.8 points difference.

First, we compared the smartphone and traditional SDMT’s ability to differentiate between HV and MS (Supplementary Fig. 1). While there is an overlap in the smartphone SDMT results between HV and MS (HV: 23.5–92.4; median 54 points; MS: 6.0–76.5; median 34.8), the Wilcoxon rank-sum test presents convincing evidence that the performance of HV and MS cohorts differ (*p* < 0.0001, CI: [13–23 points difference], (Fig. 2a).

Next, we asked if the smartphone SDMT is directly comparable to investigator administered, written SDMT. While both SDMTs show strong evidence of association (*R*^2^ = 0.71, CCC = 0.69, CI [0.62, 0.75], *p* < 0.0001; Fig. 2b), Bland-Altman plots identified systemic bias favoring written test by 7.8 points (Fig. 2c).

There are several possible sources of this bias, some deliberately addressed in the smartphone SDMT design: (1) Memorization of the symbol-digit code: Because most of our patients have experienced traditional SDMT before, they may have memorized the symbol-digit code. (2) The traditional SDMT copyrighted by Western Psychological Services facilitates performance as the first 26 items on the scoring sheet originate only from the first 6 symbols (out of 9) in the symbol-digit key^5^. Consequently, analogous bias was identified in previous SDMT comparisons^8,9^.

Because the 7.8 points bias was identified across all SDMT performance levels, adding 7.8 points to smartphone scores compensated for design differences between two SDMT tests, increasing the CCC to 0.84 (CI [0.79,0.88])^10^ (Fig. 2d).

In the MS Outcome Assessment Consortium (MS-OAC) analysis of *oral* SDMT^5^, the test-retest reliability was assessed by correlation coefficients, which ranged from 0.76 to 0.97. Therefore, we assessed test-retest reliability of *written* and *smartphone* SDMT in our MS#1 cohort when SDMT was measured six months apart. This revealed Rho = 0.95 (CI: [0.92, 0.96]), *R*^2^ = 0.90, *p* < 0.0001 for *written* and Rho = 0.87 (CI: [0.79, 0.92]), *R*^2^ = 0.80, *p* < 0.0001 for *smartphone* SDMT. We conclude, that despite randomization, test-retest reliability of smartphone SDMT is comparable to the published *oral* SDMT data and slightly lower than the non-randomized *written* SDMT.

### Smartphone SDMT highly correlates with cognitive abilities

The stronger test-retest reliability of the *written* SDMT may reflect unwanted learning effect, effectively representing a “noise” in test measurement. To formally test this hypothesis, we compared the construct and predictive validity of *smartphone* and *written* SDMT. We included an alternative cognitive test, PASAT-3, which is included in MSFC as a gold-standard (Supplementary Figs. 2–4).

**Fig. 3:**
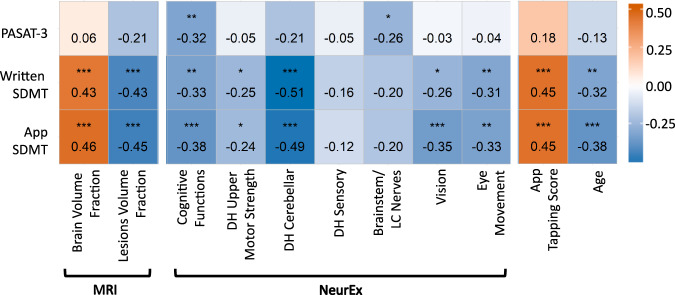
Spearman correlation coefficients between cognitive tests and outcomes from brain MRI, NeurEx features, smartphone tapping test, and age (HV = 12; MS = 112). p-values in the matrix are indicated as followed: *p < 0.01, **p < 0.001, ***p < 0.0001. Smartphone and written SDMT show evidence of correlation to the same set of features. PASAT-3 correlates with NeurEx cognitive and brainstem functions but does not correlate with volumetric MRI features.

First, we tested correlations of all three tests with the cognitive subdomain of neurological examination measured by NeurEx (Fig. 3). NeurEx is a freely-available iPad app that digitalizes neurological examination by allowing clinicians to conveniently document the severity and spatial distribution of neurological deficits using an intuitive touch interface^11^. NeurEx automatically computes major disability scales used in neuroimmunology, providing neurological subsystems and limb-specific disability data for research applications.

While all three tests correlated with the NeurEx cognitive subdomain, the smartphone SDMT achieved the highest correlation coefficient (Rho = −0.38, CI: [−0.52, −0.21], *p* < 0.0001; Fig. 3).

NeurEx also identified contributions of remaining neurological functions to cognitive test performance. As expected for an oral test not dependent on eye or motoric functions, PASAT-3 did not correlate with any other NeurEx subsystem, except with the brainstem/lower cranial nerve subscore that includes hearing and articulation functions (Rho = −0.26, CI: [−0.42, −0.09], *p* < 0.01). In contrast, both SDMTs exhibited significant correlations with dominant hand (DH) cerebellar functions (written Rho = −0.51, CI: [−0.63, −0.37], *p* < 0.0001; smartphone Rho = −0.49, CI: [−0.61, −0.34], *p* < 0.0001), vision (written Rho = −0.26, CI: [−0.42, −0.09], *p* < 0.01; smartphone Rho = −0.35, CI: [−0.50, −0.19], *p* < 0.0001), and eye movements (written Rho = −0.31, CI: [−0.46, −0.14], *p* < 0.001; smartphone Rho = −0.33, CI: [−0.48, −0.16], *p* < 0.001). As would be expected from the lesser motoric demand of touching a screen versus writing numbers, the smartphone SDMT had slightly weaker correlation with DH cerebellar functions than written SDMT. On the other hand, smartphone SDMT correlated stronger with vision and eye movements, indicating a more demanding hand-eye coordination.

We conclude that NeurEx correlations reliably identified neurological domains contributing to the performance of each test.

### Smartphone SDMT correlates with brain atrophy and MS lesions volume

Criterion validity of the traditional *oral* SDMT, preferred in MS research, was assessed in the MS-OAC by correlation with MS-related CNS tissue destruction^5^, specifically the lesion burden (T2LL) and brain atrophy (BPFr)^12^; therefore, we assessed analogous correlations.

While PASAT-3 did not correlate significantly with these MRI measures, smartphone SDMT had slightly stronger correlation coefficients in comparison to traditional written SDMT: Rho 0.46 versus 0.43 for BPFr and Rho −0.45 versus −0.43 for T2LL (Fig. 3)

Because the criterion validity of *oral* SDMT against MRI measures of MS-related brain tissue destruction was also assessed by linear regression models against multiple MRI features, we performed comparable analyses using elastic net (EN) regression. In the EN models, the BPFr and T2LL were predictor variables, while any NeurEx factor which showed strong evidence of correlation with SDMT results (including age) were included as covariates. Because complex models are susceptible to overfitting (i.e., the algorithms can use measurement “noise” to achieve better than biologically plausible results), their validity must be tested in an independent cohort. Therefore, we randomly divided our MS cohort to training (2/3) and validation (1/3) subcohorts (Supplementary Fig. 5; elastic net diagnostic plots available in Supplementary Fig. 6).

**Fig. 4:**
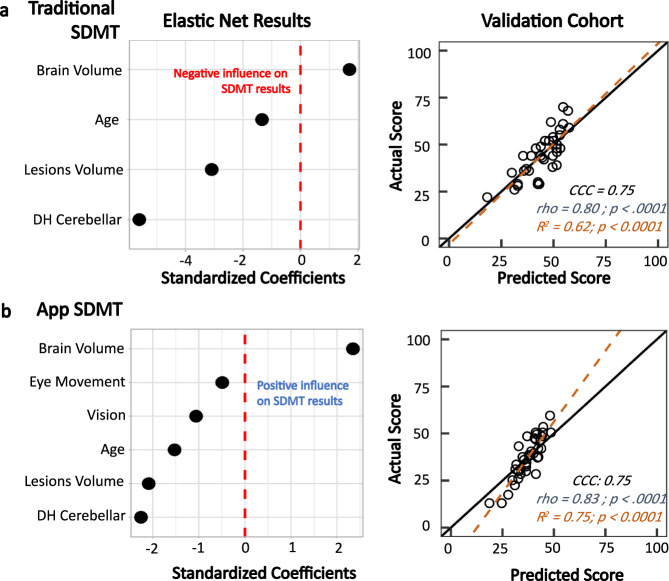
Clinical associations for traditional/written and app SDMT (HV = 12; MS = 112). **a** Elastic net regression shows that when controlling for relevant motoric factors and age, brain parenchymal fraction and T2 lesions load are highly associated with the written results. These factors predict a traditional SDMT score that agrees with the actual score at a CCC of 0.75. **b** For the app SDMT, vision and eye movement also influenced the results. Controlling for these factors, brain parenchymal fraction and T2 lesions load are highly associated with the app results. This model produces a predicted app SDMT value that agrees to the actual value at a CCC of 0.75.

In the *training cohort*, the EN models showed that BPFr and T2LL were highly associated with the smartphone and written SDMT results (Fig. 4a). While DH cerebellar function, motoric strength, and age were important covariates for both tests, the EN deemed vision and eye movement dysfunctions to be important only in the smartphone SDMT results, consistent with stronger Spearman correlations observed for app-SDMT (Supplementary Fig. 7).

When we applied resulting equations to the independent *validation cohort*, they predicted the smartphone results slightly more strongly (*R*^2^ = 0.75, Rho = 0.83, CI: [0.69, 0.91], *p* < 0.0001) than the written SDMT (*R*^2^ = 0.62, Rho = 0.80, CI: [0.64, 0.89], *p* < 0.0001; Fig. 4).

### Controlling effect of motor disability on SDMT using tapping scores

The modeling introduced in the previous section relied on clinician-derived disability measures which will not be available when administering smartphone tests at home. Thus, we asked whether we can use a surrogate to the relevant clinician-derived measures from NeuFun. Although we currently do not have enough data for app-based visual outcomes, we do have available DH tapping results, previously validated against clinician-derived cerebellar and motoric disability scores that underlie DH dexterity (ref. ^3^ and Fig. 3).

**Fig. 5:**
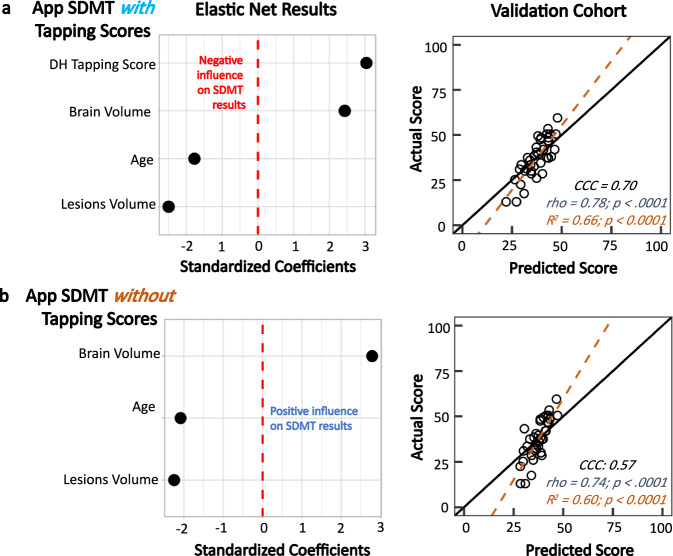
App tapping score serving as a covariate in the elastic net model (HV = 12; MS = 112). **a** Elastic net regression results with NeurEx cerebellar functions and upper motor strength being replaced by the app tapping score as a covariate. This model produces scores that agree with the actual scores at a CCC of 0.70. **b** Removing the tapping scores reduces the CCC to 0.57 and Rho to 0.74, showing that while tapping scores, like cerebellar and upper motor functions, have small influences on the app SDMT comparing to the MRI variables, it is important to include this covariate in the model.

Consequently, we asked whether this digital biomarker of DH dexterity (i.e., DH taps) can replace the neurologists scores in EN models. When we replaced the NeurEx scores of DH cerebellar and upper motoric strength with tapping results in the smartphone SDMT EN model, the model validated only slightly worse than the model that included clinicians’ data (*R*^2^ = 0.66, *p* < 0.0001; Rho = 0.78, CI: [0.60, 0.90], *p* < 0.0001; Fig. 5a).

To prove that this mathematical adjustment of measured DH disability enhances criterion validity of smartphone SDMT, we eliminated the tapping score from the model (Fig. 5b) and observed decrease in model performance. We conclude that the smartphone tapping score is a reasonable proxy for clinician-derived disability scores to control for loss of DH dexterity.

Rewriting the validated EN linear model equation and adjusting the app based SDMT result using the following formula will produce a digital biomarker that explains 66% of the variance associated with MS-related brain damage:

1 $$\begin{array}{l}55.5 \times {\mathrm{BPFr}} - 306.5 \times {\mathrm{T2LL}} = {\mathrm{App}}\,{\mathrm{SDMT}} - 0.24 \times {\mathrm{DH}}\,{\mathrm{Taps}} + 0.16 \times {\mathrm{Age}} + 5.9\end{array}$$

### Individual learning effect quantifiable with non-linear regression

A subgroup of our MS patients uses the smartphone SDMT from home on a weekly basis. This presents a unique opportunity to investigate the prevalence of practice effect. Automated identification of the time when the practice effect stops will generate a strong “baseline” against which a true progression of cognitive disability can be identified.

**Fig. 6:**
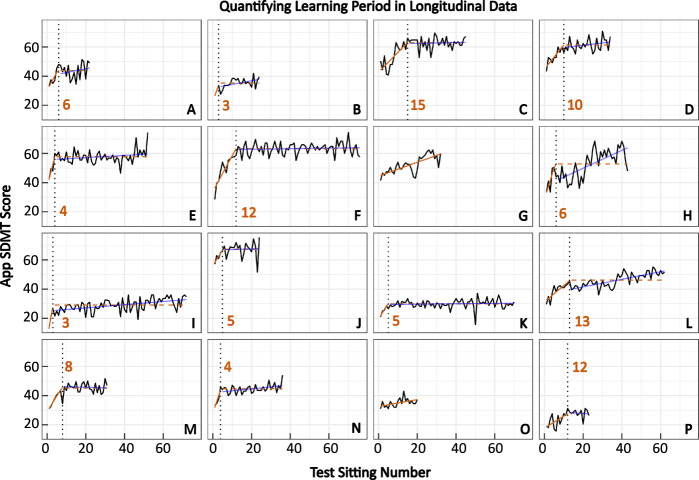
Nonlinear regression can be used to identify the inflection point in longitudinal data where learning stops occurring. Out of 16 participants (denoted by A–P; 1 HV; 15 MS) with longitudinal data (≥20 test sittings), nonlinear regression was able to identify the inflection point in 14 individuals. The solid orange line indicates the period where the individual is still learning. The dotted black line signifies the point learning inflection point that the algorithm identified. The dashed orange line denotes the algorithm’s assumption of the data’s regression line after the learning period. The solid blue line represents the actual regression line of the data after the learning period. On average, learning stops occurring after eight sittings.

The individuals’ longitudinal data showed strong indication of a practice effect; first increasing and then stabilizing SDMT answers into a plateau that fluctuates around a mean score (Fig. 6). The inflection point between the learning period and the post-learning stable period was identified automatically, using non-linear regression, in 14/16 (88%) individuals, at eight test sittings on average (Fig. 6A–F, H–N, P). The remaining two individuals (Fig. 6G and O) might be experiencing a learning effect beyond 20 repetitions due to a continuously positive slope (i.e., continuously increasing SDMT results).

### Smartphone SDMT scores have strong intra-individual reliability

Because traditional SDMT is not measured weekly, as performed in our longitudinal cohort, we re-assessed test-retest reliability of weekly longitudinal data.

**Fig. 7:**
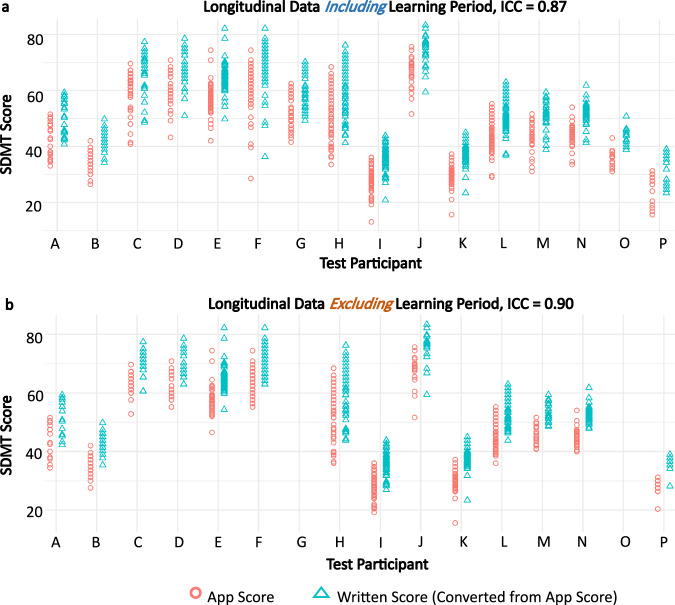
Longitudinal data exhibits good reliability. **a** With the learning period included, all longitudinal data has an intraclass correlation coefficient (ICC) of 0.87, which means that data for the same individual tend to cluster highly with each other; therefore, the data has good intra-individual reliability. **b** Without the learning period included, the ICC value increases to 0.90. In both panel **a** and **b**, the written SDMT scores (converted by adding 7.8 points to all app SDMT scores) have differences that span much larger than the current clinically significant change threshold of 4 points.

A mixed model intraclass correlation coefficient (ICC) analysis showed that in all the longitudinal data, including the “learning” period, between-patient data clustering accounted for 87% of the total variance in the smartphone SDMT score (ICC = 0.87, Fig. 7a). Post-learning, the ICC value increased to 0.90 (Fig. 7b), indicating excellent reproducibility of within-subject results.

We conclude that test-retest variability of the randomized SDMT is comparable to the test-retest reliability of episodic oral SDMT, based on published data.

### Defining decline in cognitive processing speed on a patient level

Digitalization of the SDMT will make this test broadly available in clinical practice. To allow correct interpretation of the observed changes in SDMT scores, it is imperative to determine the threshold of intra-individual decline in the test performance that exceeds natural variation. Such decline may signify MS attack or a progression of cognitive deficit.

Currently, a four-point decline in SDMT is considered “clinically meaningful”^13–15^. Because this cut-off was derived from group comparisons, it is unclear whether it applies to individual subjects.

**Fig. 8:**
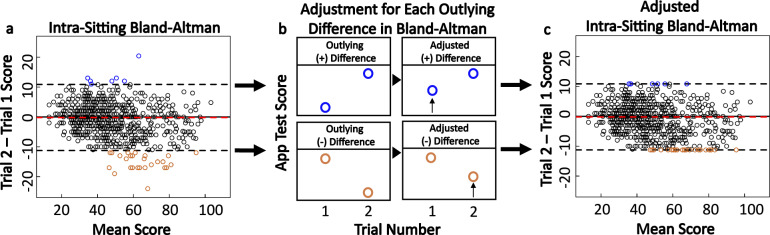
Outlier adjustment procedure for test sittings with two trials. **a** Score differences and means for test sittings with two trials are shown on a mixed-effects Bland–Altman plot (HV = 33; MS = 119). The limits of agreement are −11.24 and 10.85, and the mean difference is −0.19. Sittings with differences that fall outside of the limits of agreement, indicated by blue and orange points, are considered to have one trial that is an outlier. **b** In the sittings with outlier trials, the lower performing trial’s score is increased to reach the limits of agreement thresholds. **c** The original mixed-effects Bland–Altman plot, now shown with the outlying differences after score adjustment.

We already demonstrated that the test-retest reliability of either SDMT yielded comparably strong correlation coefficients to published data for oral SDMT. However, this excellent test-retest reproducibility on a group level translated to intra-individual variance much greater than four points (Fig. 8a). Likewise, in MS subjects who perform SDMT weekly at home, we observed intra-individual variability greatly exceeding four SDMT points (Fig. 7), even when measured in the post-learning period. None of these longitudinal testers reported MS relapse or significant deterioration of their neurological functions.

**Fig. 9:**
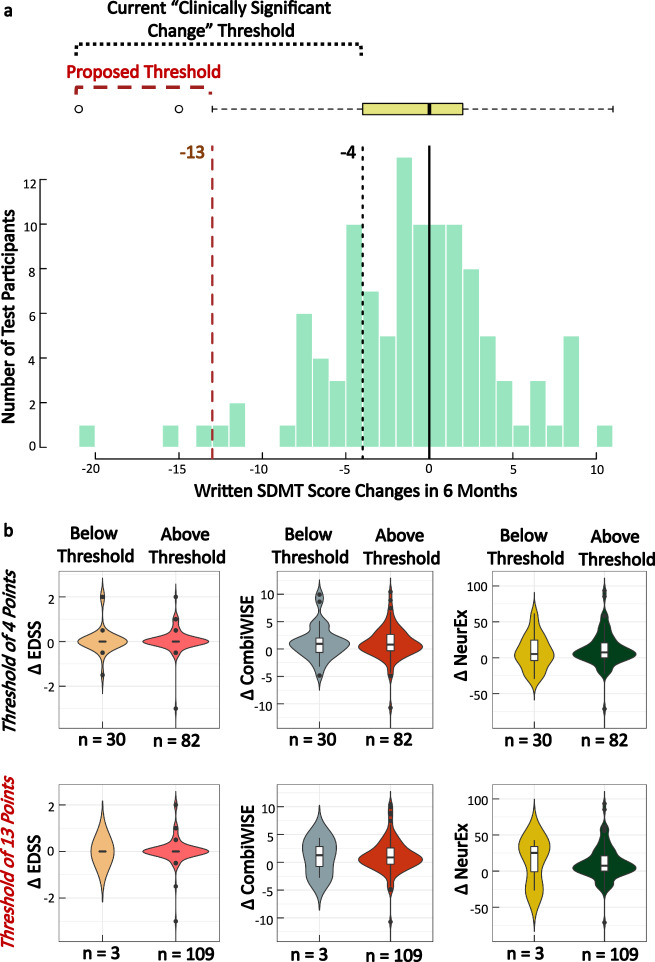
Proposing a new change threshold for the written SDMT. **a** Distribution of written score differences for participants who completed the written SDMT in the course of six months. Using the current score decrease of four points, approximately 24% of participants would have been falsely classified as having meaningful decline in their cognitive processing speed. We propose that this threshold should be increased to 13 points, or (average difference) – (1.5 × IQR of the differences). **b** Comparison of changes in disability scores for individuals who are above or below the defined threshold. At the four-points threshold, there is no significant difference in disability score changes between individuals who are below and above the threshold. At the 13-points threshold, while there is also no significant difference in disability changes between individuals who fall above or below the threshold, the disability changes are much larger in CombiWISE and NeurEx. The lack of statistical significance may be due to the low number of individuals who fall below the 13-points threshold.

Next, we plotted the intra-individual differences between two traditional written SDMT tests collected six months apart (Fig. 9a, Table 2); we observed Gaussian distribution with mean difference of −0.7 and two standard deviations of 10.3, analogous variance measured in the test-retest Bland–Altman plots (Fig. 8a). We then formally tested if the four-point written SDMT decline identified change in any other clinically meaningful outcome. The publication that proposed a four-point decline in SDMT as clinically meaningful (on a group level) used decline in employment status as the outcome^14^. Because none of our MS patients experienced decline in employment status between SDMT tests measured six months apart, we asked whether the patients who declined in SDMT performance by at least four points differed in any other objective measure of disability. Since the mean/median EDSS change over six months was zero (Fig. 9b), we used disability scales with greater sensitivity, the Combinatorial Weight-Adjusted Disability Scale (CombiWISE; continuous scale ranging from 0 to 100)^16^ and the NeurEx (Continuous scale ranging from 0 to theoretical maximum of 1349)^11^.

Thirty MS patients (26.8%) fulfilled four-point SDMT decline criterion, but we observed no statistically significant differences in either CombiWISE or NeurEx disability progression between MS patients who fell below and above this threshold (Fig. 9b; ∆ CombiWISE comparison +0.9 versus +0.8: *p* = 1.0; ∆ NeurEx comparison + 5.2 versus +7.5: *p* = 0.4). As evidenced by positive numbers, in contrast to EDSS, both CombiWISE and NeurEx measured progression of clinical disability, which was equal in patients whose SDMT scores declined by at least four points and patients whose scores did not.

Next, we used a statistical definition of change that exceeds test-retest variance based on the distribution of differences in six months SDMT values: Average – 1.5 × Interquartile Range (IQR) change = 13-point decline. Only three MS subjects (2.7%) declined in SDMT performance beyond this threshold during six months follow-up. This cohort is too small to obtain reliable statistics, but the median CombiWISE and NeurEx changes were higher in this small group in comparison to the remaining 109 subjects with MS (∆ CombiWISE +1.3 versus +0.9; ∆ NeurEx + 25.3 versus +7.5).

We conclude that both written and smartphone SDMT have similar variance, requiring a difference of 13 to 14 SDMT points to identify decline that exceeds test-retest fluctuation in SDMT performance when comparing only two SDMT tests within the same subject.

**Fig. 10:**
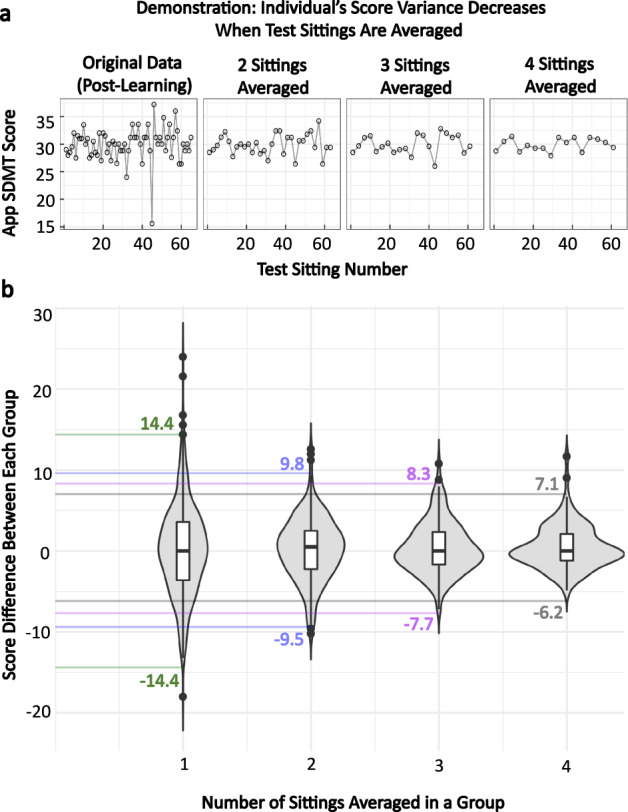
Determining the change threshold for the app based SDMT. **a** A demonstration of how the thresholds are calculated in the app SDMT. For each individual, the score differences between one sitting, two sittings averaged, three sittings averaged, and four sittings averaged were calculated. **b** Plotting these differences allows for identification of the change thresholds that should be used when the participant’s score changes are based on single test sittings or multiple sittings averaged together.

Next, we hypothesized that granular collection of data may lower this threshold of true deterioration when comparing period averages. The Gaussian distributions of test-retest data suggests random distribution of noise, that could be limited by averaging multiple weekly tests within individual patients, akin to “repeated measures”. Thus, within the granular testing cohort, we compared the variance of single adjacent tests, with an average of two, three, or four adjacent tests (Fig. 10). We observed an expected decline in variance; from 14.4 SDMT points for single test comparisons; to 9.5 points for average of two adjacent tests; 7.7 points for average of three adjacent tests; finally, 6.2 points for average of four adjacent tests.

We conclude that when a subject performs SDMT only twice, the decline of more than 14 points reliably exceeds natural variability in the test performance and should trigger a search for structural cause, such as MS exacerbation of progression of cognitive deficit. Lower level of SDMT performance decline should trigger repeated testing. In this regard, granular collection offers advantage over episodic collection of SDMT data, as averaging multiple test repetitions lowers the threshold for identifying true decline in test performance in individual subjects.

## Discussion

Neurological examination is a powerful tool to identify, localize, and grade neurological deficits. However, economic pressures limit access to this essential tool worldwide^1^. Because this situation is unlikely to improve, it is imperative to find alternative reliable measurements of neurological disability. The digital health revolution spurred development of “medical” apps. However, a recent conference on digital health, *Digital Health: From Science to Application* (Perakslis, E.D., Coravos, A., MacRae, C.A. & Godfrey, A.) concluded that technical development of these apps is easy in comparison to validating their value for intended use, in accordance with FDA guidance^17^. Consequently, most medical apps are released without assessment of their psychometric properties^18^.

To assure broad use of NeuFun, we focused our developmental efforts on tests that can be self-administered on smartphones instead of tablets due to the broad availability of the former, including in developing countries. This would keep the cost of the test low and allow telemedicine with home administration of the test. However, the adaptation of paper-based tests to much smaller smartphone screens requires greater involvement of visual system/hand-eye coordination (Figs. 3, 4). This shortcoming likely underlies the slightly lower agreement with traditional SDMT observed in our study, as compared to the digitalized oral SDMT in the Cognitive Assessment for Multiple Sclerosis (iCAMS), which is tablet-based and uses a trained administrator^19^.

As the published MS-OAC developed an excellent framework for validating the oral SDMT^5^, we followed this framework to validate smartphone SDMT. Despite randomization, smartphone SDMT has comparable discriminative, psychometric, construct, predictive, and criterion validity to published parameters of oral SDMT. In addition, previous publications on oral SDMT often excluded subjects with hearing/speaking disabilities and with comorbidities such as alcoholism and depression to limit noise in SDMT performance^14^. This over-estimates the psychometric validity of oral SDMT compared to routine practice. We did not exclude any MS patients based on disability or comorbidities. We also blinded divergent assessments (i.e., clinicians scores, SDMT, and MRI measures), limiting bias in assessing psychometric characteristics of both SDMT tests.

The touch-based smartphone SDMT adaptation has predicted limitations in that motoric disability of the performing hand affected SDMT performance. However, as illustrated, careful mapping of contributing neurological functions permits mathematical adjustment for measured disabilities as covariates to isolate the tested neurological domain. Using this methodology, the smartphone SDMT results, when adjusted for visual disability and DH dexterity, explained a stunning 75% of the variance in the EN linear regression model against a composite of brain atrophy and T2LL in the independent validation cohort. This remarkable criterion validity compares favorably to oral SDMT in MS (i.e., Rho = 0.83 in this study versus Rho = 0.71 in published study^5^) and validates the ability of smartphone SDMT to reliably measure MS-related brain injury.

Additional advances of the test digitalization are algorithmic identification of the learning and granular longitudinal sampling permissive to averaging temporally adjacent results; this provides an accurate “baseline” and lowers threshold for identifying true (i.e., disease-related) decline in test performance. Indeed, our use of non-linear regressions yielded an identical result (i.e., stabilization of the learning effect after 8 trials) previously reported in natalizumab-treated MS patients^20^.

Thus, our paper demonstrates that, in principle, SDMT is adaptable to self-administered digital tests that may be broadly utilized in neurology practices and guide therapeutic decisions. This brings an associated challenge to correctly interpret the test results. In this regard we acknowledge that our HV data are still limited and need to be expanded to provide reliable normative data across all age-groups. The other problem we addressed is the threshold of decline in SDMT performance that exceeds natural variation and therefore may represent actionable change due to e.g., MS relapse or disease progression. We demonstrated that this threshold is quite large, representing over 14-point decline when the subject performs the test only twice, which can be lowered to 7-point decline if the person performs the test four times and averages the test performance. Because these data-driven, intra-individual thresholds exceed currently accepted four-point decline in SDMT performance identified on a group level as clinically meaningful, we want to clarify the reasons for this apparent discrepancy.

The paper that suggested four-point decline in SDMT as “clinically meaningful” studied retrospectively two groups of MS patients: those who experienced decrease in employment status, and those who did not^14^. These MS patients had a battery of neurocognitive tests twice, on average 2-4 years apart. From ten cognitive tests administered, only the change in SDMT differentiated the two MS groups with statistical significance: the subjects whose vocational status declined showed decline on SDMT by 3.0 +/− 8.0 points, while patients with a stable vocational status showed SDMT improvement of 1.3 +/− 6.6 points. We note that SD of intra-individual SDMT changes measured in this highly pre-selected MS cohort still greatly exceeds four-point threshold. Considering the accepted cut-off of 2 SD to differentiate true biomarker change from random test variation, even this cohort generates a threshold of 13–16 SDMT points decline on the individual level. The papers that corroborate the four-points SDMT decline threshold on a group level did not measure test-retest variance on an individual level^13–15^ and did not measure the predictive power of four-point SDMT decline on vocational status of MS patients in an independent, prospective MS cohort.

Thus, the apparent discrepancy of the conclusions between this and previously published papers resides in the way this “clinically-meaningful” threshold was applied; the previous studies applied this threshold on a group level, whereas we sought to determine the threshold that can alert the subjects and their clinician to the actionable intra-individual decline in test performance. Indeed, four-point SDMT decline on a group level should be deemed clinically-meaningful, as longitudinal MS studies lasting less than three years did not measure any decline in SDMT performance (summarized in ref. ^5^). Finally, we determined only the threshold of decline in the intra-individual performance that exceeds natural variation; we had too few MS patients who exceeded this threshold during the follow-up period to determine the “clinical significance” of such decline.

Unfortunately, interpreting the results correctly is not the only challenge in translating a smartphone-based test to clinical practice. These tests represent medical devices, subjected to regulation by regulatory agencies. The psychometric properties of the smartphone medical device are linked to a specific hardware/software combination. Being mindful that different smartphone sensors can influence NeuFun results, we used only two comparable devices and avoided vector-based rescaling of graphics to different screens. However, test commercialization requires continuous assessment and validation that no future software or hardware changes alter psychometric properties of the test or the established protocols that assure automated, reliable, and private communication between patient/device and the clinician’s office.

In conclusion, smartphone adaptation of the SDMT accurately measures reaction time in a patient-autonomous manner and provides several advantages against investigator-administered, not digitalized, SDMT. Rapid development and validation of the entire NeuFun suite will facilitate tele-neurology, making neurological care more accurate and less expensive.

## Methods

### App development

The smartphone SDMT was written in Kotlin and Java using the latest Android Studio integrated development environment. The test is delivered as an Android package (APK), and the results are stored in a secured online database under alphanumeric code that lacks personal identifiable information (PII). The smartphone SDMT uses the Android operating system (Android 9 and up), with graphics optimized for only two types of smartphones used for all subjects (Google Pixel XL/2XL) in colorblind-friendly visualizations. To assure equivalence of the test between the smartphones, no vector-based rescaling was used. Instead, the size and location of the graphics is predefined and displaced equivalently on different phones. The SDMT symbols are Unicode characters.

### Participants

This study was approved by and carried out in accordance with the recommendations of the Central Institutional Review Board of the National Institutes of Health (NIH). All subjects gave a written or digital informed consent in accordance with the Declaration of Helsinki.

Healthy volunteers (HV) and MS participants enrolled in the protocol: Comprehensive Multimodal Analysis of Neuroimmunological Diseases in the Central Nervous System (Clinicaltrials.gov identifier NCT00794352).

Two HV cohorts were recruited: *#1. HV who underwent all study procedures* (including neurological examination, PASAT-3, traditional SDMT, MRI of the brain, lumbar puncture [LP]; 16 HV total). The HV inclusion criteria are: (1) At least 18 years old; (2) Normal vital signs; (3) Able to give informed consent; (4) Able to undergo all research procedures. HV exclusion criteria were: (1) Systemic inflammatory disorder, or inflammatory or non-inflammatory neurological diseases; (2) Previous or current history of alcohol and substance abuse; (3) Contraindications to associated procedures.

Because this number of HV is understandably limited, we recruited a second HV cohort solely to obtain normative NeuFun data: Smartphone app *HV group (#2) uses the NeuFun remotely to mimic a real-world situation* (*n* = 27). To mimic the population that would provide normative data via the App marketplace, this sub-study collects no personally identifiable information (PII) and poses no inclusion/exclusion criteria. Subjects are self-declared to be healthy and sign digital informed consent that tested their comprehension of the testing instructions and associated risks/benefits directly via smartphone. They then input their age (in years), gender, and take the test under assigned alphanumeric code. Because no systemic differences were identified between HV cohorts #1 and #2 (not shown), the two HV cohorts were merged for the analyses.

Inclusion criteria for patients in the NCT00794352 protocol are: (1) Clinical syndrome consistent with immune-mediated CNS disorder; and/or (2) Neuroimaging evidence of inflammatory and/or demyelinating CNS disease; (3) Age ≥12 years; (4) Adults that are able to give informed consent, or minors with a parent or legal guardian able to consent, with child willing to assent; (5) Able to undergo all research procedures. The exclusion criteria included: (1) Medical condition that would make participation impossible or risky; (2) Medical contraindications for MRI; (3) Unwilling to consent for collection of biological samples. The diagnosis of MS was based on 2010 and (after 2017) 2017 McDonald’s MS diagnostic criteria^21,22^.

Paralleling the two HV cohorts, we also have two cohorts of MS patients: *#1. MS patients tested only in the NIH clinic, on average every 6–12 months (n* *=* *154)*. These patients had neurological examinations and a brain MRI within 1–48 h of the NeuFun. Clinician documented neurological examinations in NeurEx, an iPad-based app that allows fast and intuitive documentation of neurological examination, including its spatial information, by touching human body diagrams. NeurEx then automatically calculates traditional disability scales, including Expanded Disability Status Scale (EDSS)^11^. *#2. MS sub-cohort with granular NeuFun collection at home (n* *=* *15)*. These MS patients expressed interest, were given a smartphone pre-loaded with NeuFun, and were asked to test at least once per week around the same time of day. Subjects who completed at least 20 sittings from MS#2 and HV#2 cohorts comprise the longitudinal/granular data. The first test sitting from subjects in all cohorts comprised the cross-sectional data.

Participant demographics are summarized in Tables 1–2 and the contribution of different cohorts to presented data is summarized in the Supplementary Figs. 8 and 9.

**Table 1:** Demographics table with baseline information for cross-sectional data.

a. Demographics	All cross-sectional smartphone participants (*N* = 193)			
	PPMS (*n* = 54)	SPMS (*n* = 42)	RRMS (*n* = 58)	HV (*n* = 39)
Age (year)				
Mean ± SD	58.7 ± 11.1	58.3 ± 10.3	47.3 ± 9.6	41.1 ± 14.0
Range	18.5–72.8	33.0–75.5	30.2–77.2	21.5–67.9
Gender (% of cohort)				
Female	56	57	60	59
Male	44	43	40	41

b. Demographics	Cross-sectional smartphone participants with clinical data (*N* = 112)			
	PPMS (*n* = 46)	SPMS (*n* = 26)	RRMS (*n* = 40)	HV (*n* = 12)
Age (year)				
Mean ± SD	58.5 ± 10.8	56.7 ± 10.8	45.6 ± 8.3	48.9 ± 6.8
Range	18.5–72.8	33.0–75.5	30.2–66.4	40.1–60.8
Gender (% of cohort)				
Female	61	58	62	75
Male	39	42	38	25
NeurEx				
Mean ± SD	162.0 ± 61.0	164.0 ± 60.1	68.5 ± 48.3	18.4 ± 15.1
Range	34.2–312.0	67.1–334.0	0.0–216.0	0–54.2
EDSS				
Mean ± SD	5.6 ± 1.0	5.7 ± 1.0	3.6 ± 1.5	1.9 ± 0.8
Range	3.0–7.0	4.0–6.5	0.0–6.5	0.0–3.0

**Table 2:** Demographic table with information for individuals used in written SDMT change threshold determination and app SDMT longitudinal analyses.

a. Demographics	Participants included in written threshold analysis (*N* = 112)			
	PPMS (*n* = 47)	SPMS (*n* = 33)	RRMS (*n* = 32)	
Age (year)				
Mean ± SD	57.3 ± 11.5	57.0 ± 8.2	47.2 ± 10.8	
Range	19.0–70.8	38.1–71.2	29.8–77.7	
Gender (% of cohort)				
Female	60	67	62	
Male	40	33	38	
NeurEx				
Mean ± SD	159.0 ± 69.9	178.0 ± 48.2	64.8 ± 44.5	
Range	54.3–328.0	74.4–288.0	4.2–165.0	
EDSS				
Mean ± SD	5.7 ± 1.1	6.1 ± 0.8	3.5 ± 1.5	
Range	2.5–7.5	4.0–7.5	1.0–6.5	

b. Demographics	Longitudinal smartphone participants (*N* = 16)			
	PPMS (*n* = 8)	SPMS (*n* = 5)	RRMS (*n* = 2)	HV (*n* = 1)
Age (year)				
Mean ± SD	59.0 ± 12.9	59.5 ± 4.9	54.1 ± 1.3	63.1
Range	28.2–69.3	54.2–64.9	53.2–55.0	Na
Gender (% of cohort)				
Female	38	40	0	100
Male	62	60	100	0
NeurEx				
Mean ± SD	197.0 ± 112.0	164.0 ± 103.0	46.1 ± 18.2	Na
Range	88.9–436.0	67.1–334.0	33.2–59.0	Na
EDSS				
Mean ± SD	6.1 ± 1.2	5.4 ± 1.0	3.0 ± 0.7	Na
Range	4.5–8.0	4.0–6.5	2.5–3.5	Na

Demographics in Table a comes from the second visit of the 6-months span. Demographics in Table b comes from the first visit (baseline) values.

### Volumetric brain MRI analyses

The details of the MRI sequences were previously published^16,23^. Locally anonymized and encrypted T1 magnetization-prepared rapid gradient-echo (MPRAGE), or fast spoiled gradient-echo (FSPGR) images and T2 weighted three-dimensional fluid attenuation inversion recovery (3D FLAIR) DICOM files were analyzed by an automated segmentation algorithm LesionTOADS^24^, implemented into a cloud service for medical image processing by QMENTA (www.qmenta.com). The sequences are anterior commissure-posterior commissure aligned, co-registered and skull stripped. The T1 image is additionally bias-field corrected. The segmentation is performed by combining a topological and statistical atlas resulting in computed volumes for each segmented tissue in mm^3^. The segmentation maps were quality checked (M.V.) with errors corrected by re-running the algorithm, or uncorrectable scans were excluded.

### Implemented measures to prevent bias

All subjects received a sequential alphanumeric identification code. NeuFun, written SDMT, and PASAT-3 were collected by investigators blinded to neurological examination and MRI results. Clinicians performing neurological examinations were blinded to MRI and NeuFun results. MRI analyses were performed by investigators blinded to clinical examination and NeuFun outcomes. All MS patients and HVs with available data in the research database were included, irrespective of level of disability or comorbidities.

### Data-driven identification of minimum test time for smartphone SDMT

**Fig. 11:**
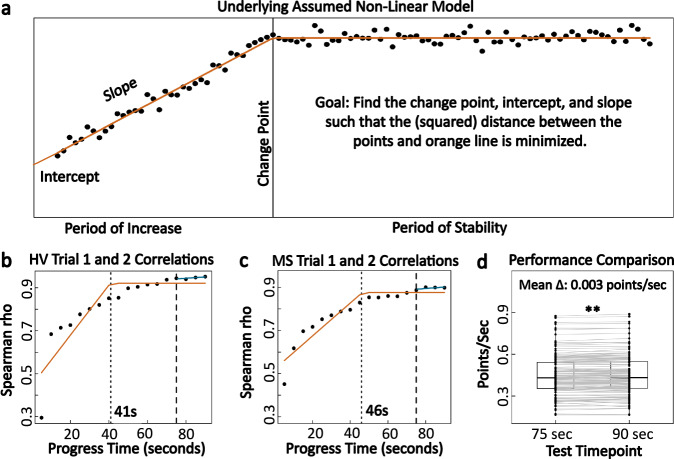
Minimum time needed for reliable app based SDMT performance. **a** Demonstration of how the non-linear regression model works to find the inflection/change points. **b, c** Plots of correlation in the scores between two trials (in the same test sitting) as the testing time progresses for HV (*n* = 22) and MS (*n* = 119). Orange lines represent the underlying non-linear regression models fitted to the data. Dotted lines indicate the minimum time needed for reliable performance based on the non-linear regression algorithm. Dashed lines point to the correlation coefficients at 75 s, or the time constraint that was eventually implemented. Blue lines indicate that after 75 s, the correlation continues to slightly improve. **d** Comparison of testing speed, in points per seconds, within the same trial at 75 and 90 s. Individuals show strong evidence of increasing their speed towards the end of the test (*p* < 0.001) but the change of 0.003 points/s is clinically insignificant.

Time is an essential determinant in the utility of a digital test: because NeuFun is designed to recreate the neurological examination, it consists of many tests. If their administration takes too long, subjects will not complete the suite. Because we could not find published rationale for the selection of 90 s for the standard SDMT, we determined the shortest SDMT duration necessary to provide reliable information using test-retest reliability data from individuals who completed two 90-s trials in the same sitting. Spearman correlation coefficients of the number of correct answers were generated for every 5 s of the duration of the two trials (i.e., 5, 10, 15 till 90 s). Expectedly, these correlations increased with the duration of the test, but eventually plateaued around Rho = 0.9 (Fig. 11). Using non-linear regression, a method which fits the data with several contiguous lines that minimize the squared distance between measured data and the regression model, we identified 2 inflection points in these time-lapse data: first at 41 s in HV and 46 s in MS, after which the correlations still improved at slower pace (Fig. 11b, c). To maximize test reliability, we selected 75 s of test duration, for at this duration the test-retest correlation has maximized.

The within-subject test performance (correct answers/sec) between 75 s and 90 s tests identified miniscule difference of 0.003 points/s (*p* < 0.001). This is clinically irrelevant compared to between participant’s variance that ranges from 0.2 to 0.9 correct answers per second (Fig. 11d).

To keep the smartphone SDMT results comparable to standard SDMT, smartphone SDMT scores were re-calculated using the formula:

2 $${\mathrm{Smartphone}}\,{\mathrm{SDMT}}\,{\mathrm{score}} = \frac{{{\mathrm{Total}}\,{\mathrm{correct}}}}{{{\mathrm{Total}}\,{\mathrm{testing}}\,{\mathrm{time}}}} \times 90$$

### Data pre-processing: identifying and adjusting outlier values

For subjects who took the test twice in the same setting (day) a Bland–Altman plot assessed test-retest bias (Fig. 8a^25^; the equations are in Supplementary Fig. 10). We observed slightly improved performance in the repeated test (i.e., learning effect, with trial 2 on average 0.19 correct answers better than trial 1 (Fig. 8a)). Surprisingly, within-subject test-retest variance greatly exceeded the 4-point difference that is currently accepted as clinically meaningful decline in SDMT performance^13–15^.

Intra-individual outliers in test-retest scores were identified as paired sittings where the difference fell outside of the Bland-Altman limits of agreement. Because the participants demonstrated ability to perform the test at the higher level, we assigned an outlier status to the lower performing score. Rather than excluding the lower-performing outlier (which would introduce bias) we increased the outlier score to the closest limit of agreement on the Bland–Altman plot (Fig. 8b, c).

### Statistics

All analyses were done in R^26^ (Supplementary Fig. 9), with packages found in the refs. ^27–46^. The comparison is based on non-parametric Wilcoxon signed-rank test (paired data) and Wilcoxon rank-sum test (unpaired data). Correlation are assessed using Spearman correlation coefficients, and associations that control for confounding variables are conducted using elastic net (EN) regression (Supplementary Fig. 5). A conservative significance threshold of 0.01 is used for all analyses.

Lin’s coefficient of concordance (CCC; 0 to 1^47^); captures the degree in which the regression line follows a 1:1 trajectory, with CCC = 1 representing 100% agreement. An intraclass correlation coefficient (ICC; 0 to 1; Supplementary Fig. 7) compares the proportion of variance between individuals to within individuals (between/ [between + within]). ICC = 1 means there is virtually no variance in scores that come from the same individual (no within-individual variance), and therefore, an individual’s test scores are consistently identical. The sitting number (test #1, 2, 3 etc.) was used as the fixed effect, as the ICC using number of days from the first trial showed analogous results (Supplementary Figs. 11–13).

Confidence intervals (CI) for all analyses are reported as 95%.

### Training and validation cohorts for elastic net (EN) modeling

In the EN regression (*glmnet* R package), the cross-sectional data was randomly divided into training (2/3) and validation (1/3) cohorts stratified for age and SDMT scores (Supplementary Fig. 14).

There is a preprint version of this paper^7^.

### Reporting summary

Further information on research design is available in the Nature Research Reporting Summary linked to this article.

## Supplementary information

Supplementary Information

Reporting Summary

## Data Availability

Data used for all analyses can be found in the Supplementary Files. Raw data can be found at: https://github.com/bielekovaLab/Bielekova-Lab-Code/tree/master/FormerLabMembers/Linh/sdmt_analyses.

## Code availability

The Android app used in this study is available to all interested research collaborators under NIAID non-commercial limited use agreement. The app was developed for Android 6 and later. The code used for data analysis is available at: https://github.com/bielekovaLab/Bielekova-Lab-Code/tree/master/FormerLabMembers/Linh/sdmt_analyses. All data analysis was done using R.
