# Supplementary material for: Smartphone-based symbol-digit modalities test reliably captures brain damage in multiple sclerosis
Source: NPJ Digit Med. 2021 Feb 24;4:36. doi: 10.1038/s41746-021-00401-y (PMC7904910; doi:10.1038/s41746-021-00401-y)
Supplement: Supplementary file 1 — Supplementary Information [file 41746_2021_401_MOESM1_ESM.pdf]

Smartphone-Based Symbol-Digit Modalities Test Reliably Captures Brain Damage in Multiple Sclerosis

Supplementary Materials

Supplementary Figure 1

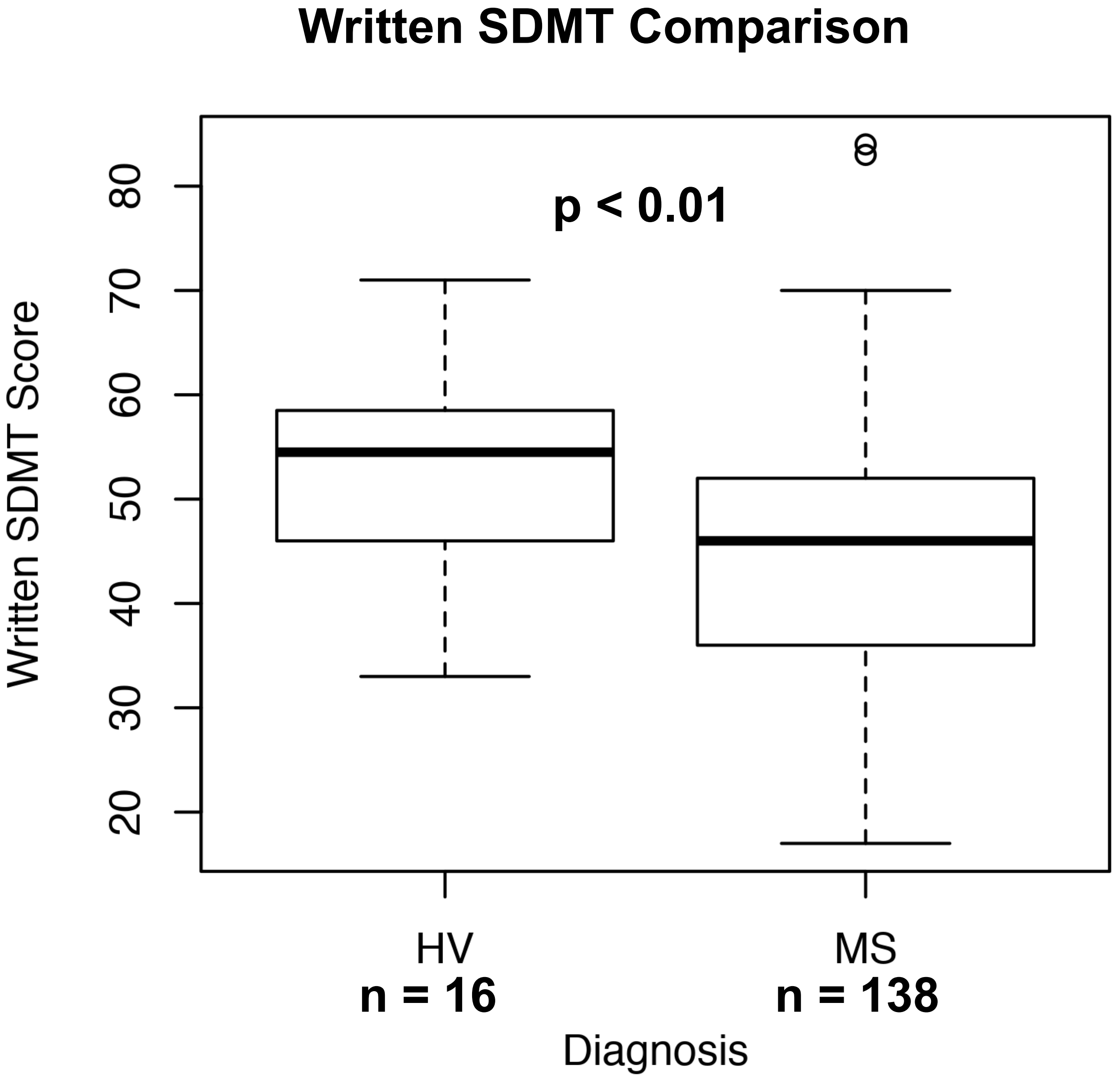

Comparison of written SDMT between healthy individuals and MS patients. Individuals came from the same cohorts documented in Figure 4 b,c. The difference between HV and MS median score is 8.5 points.

Supplementary Figure 2

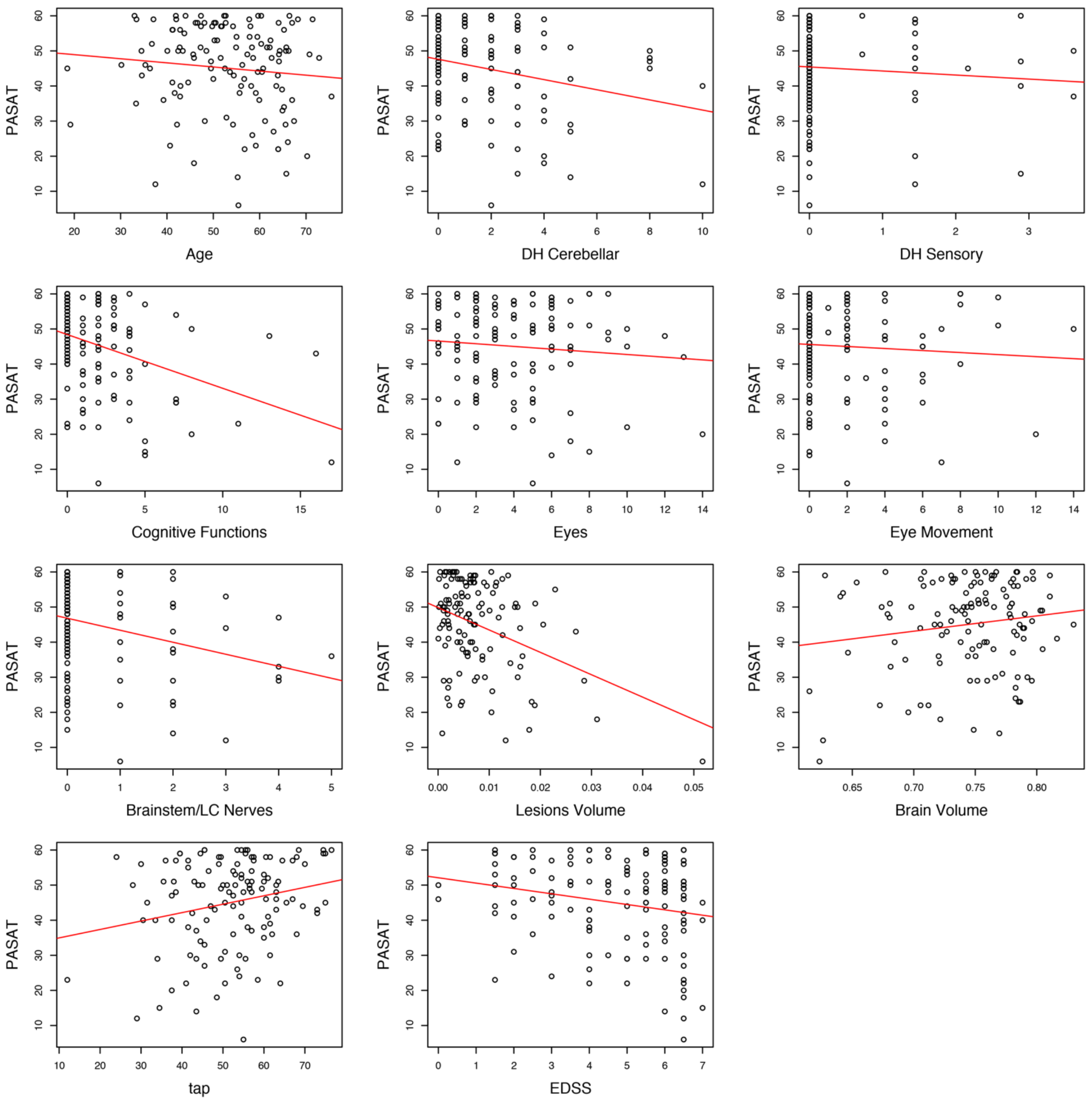

Plots of PASAT scores against clinical features, volumetric MRI, and smartphone dominant hand tapping.

Supplementary Figure 3

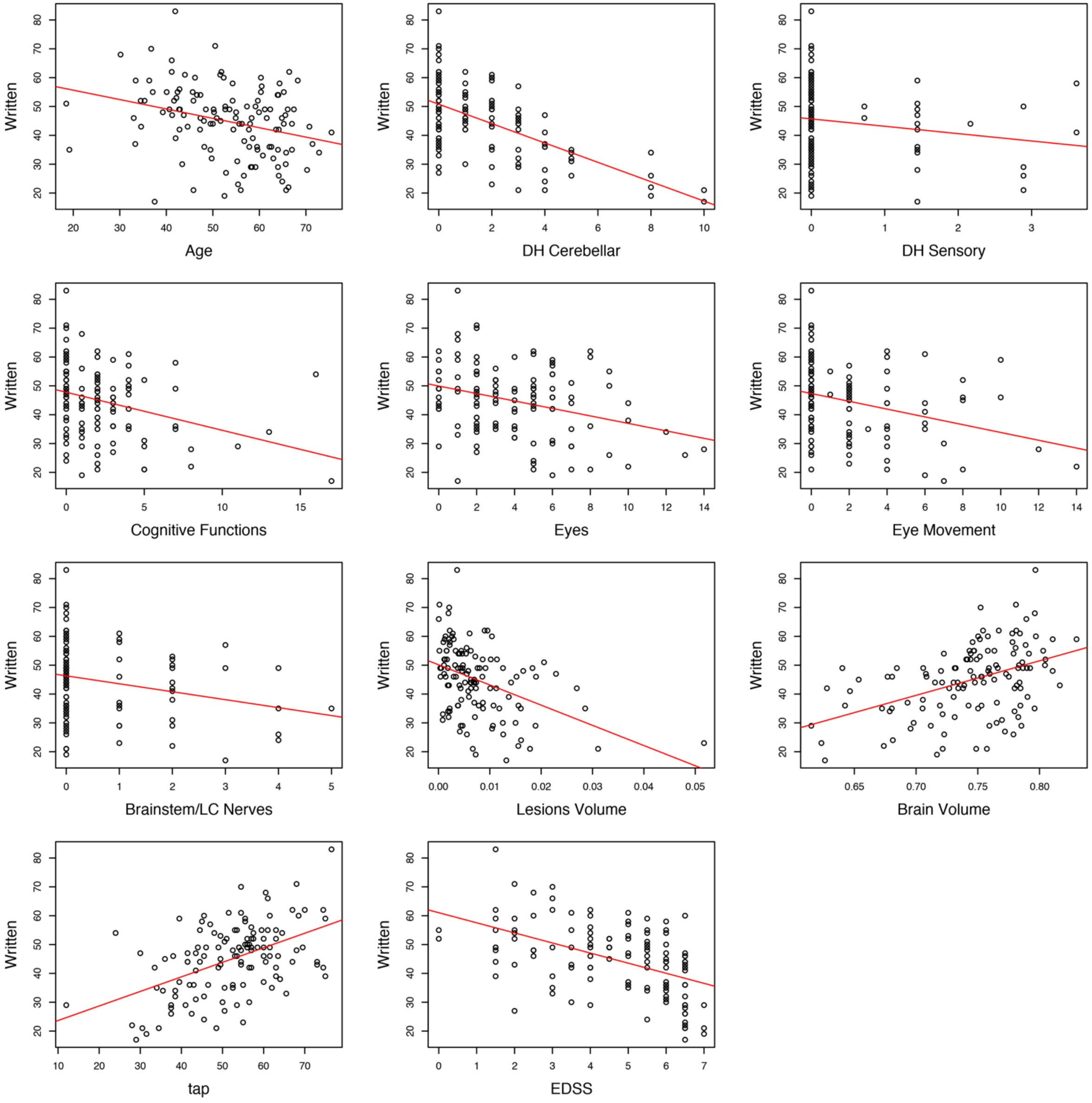

Plots of written SDMT scores against clinical features, volumetric MRI, and smartphone dominant hand tapping.

Supplementary Figure 4

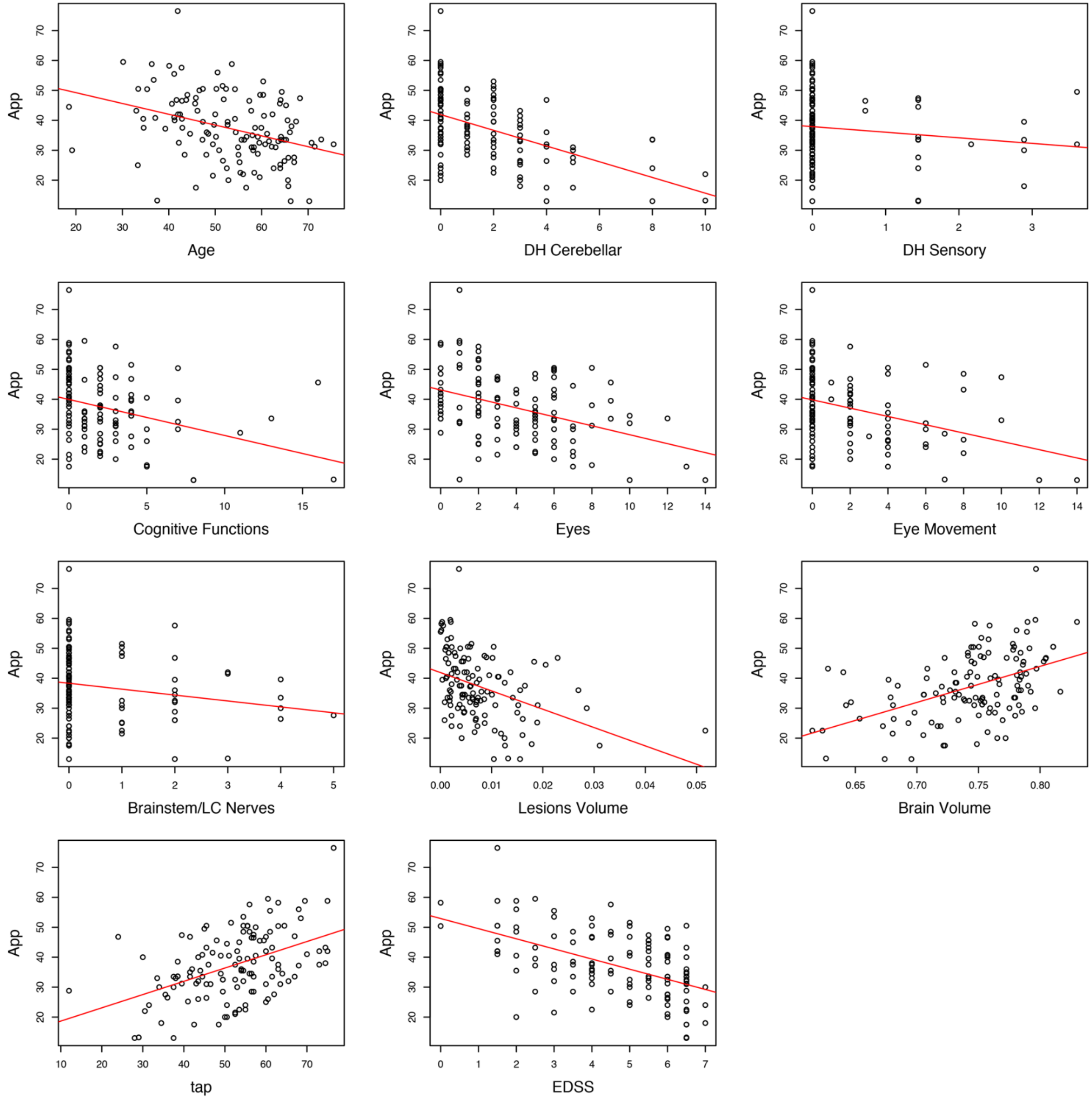

Plots of app SDMT scores against clinical features, volumetric MRI, and smartphone dominant hand tapping.

Supplementary Figure 5

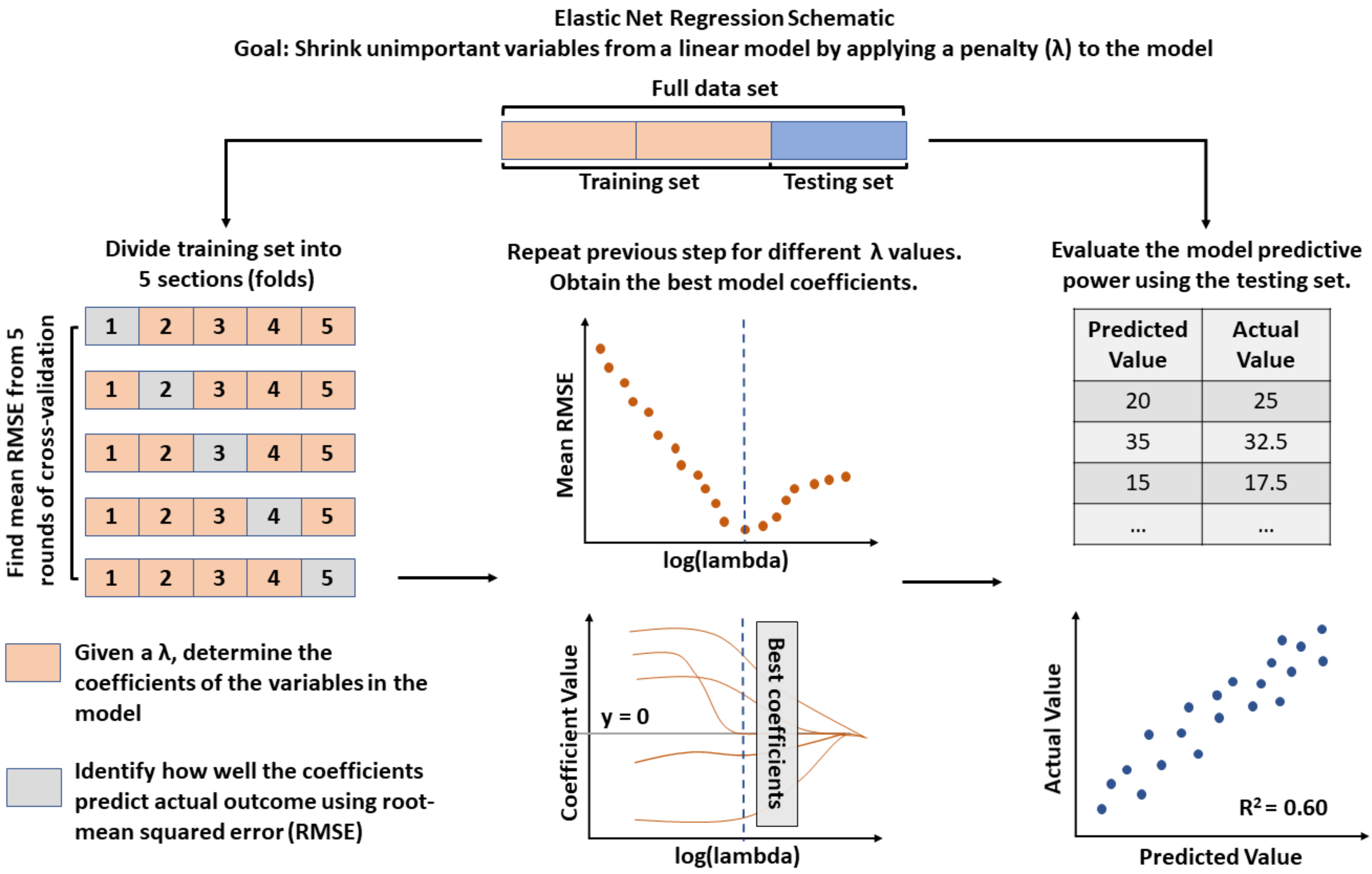

Supplementary Figure 6

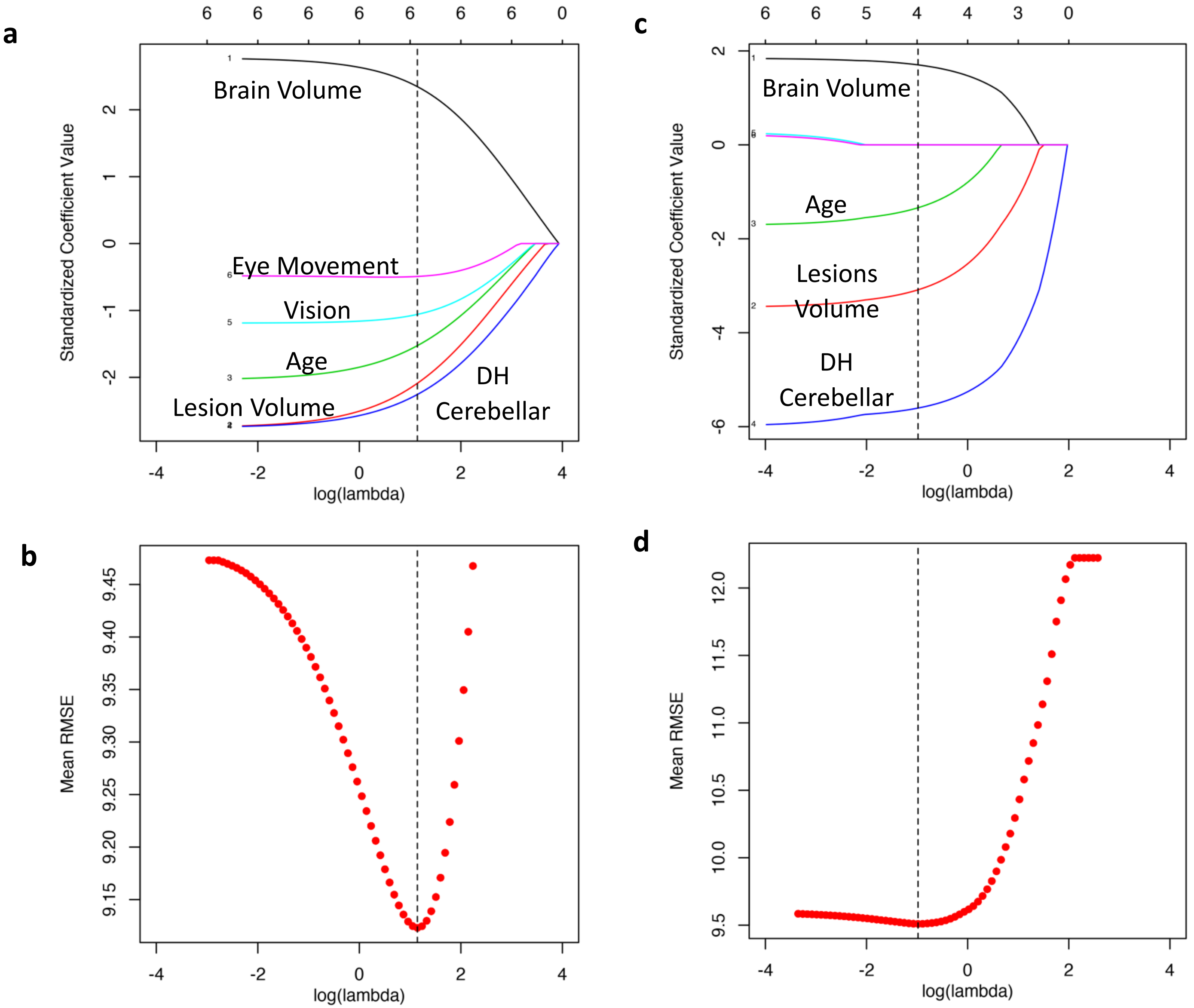

Coefficient path and mean RMSE plots for written and app SDMT elastic net regressions. **(a-b)** Plots for app SDMT. **(c-d)** Plots for written SDMT.

Supplementary Figure 7

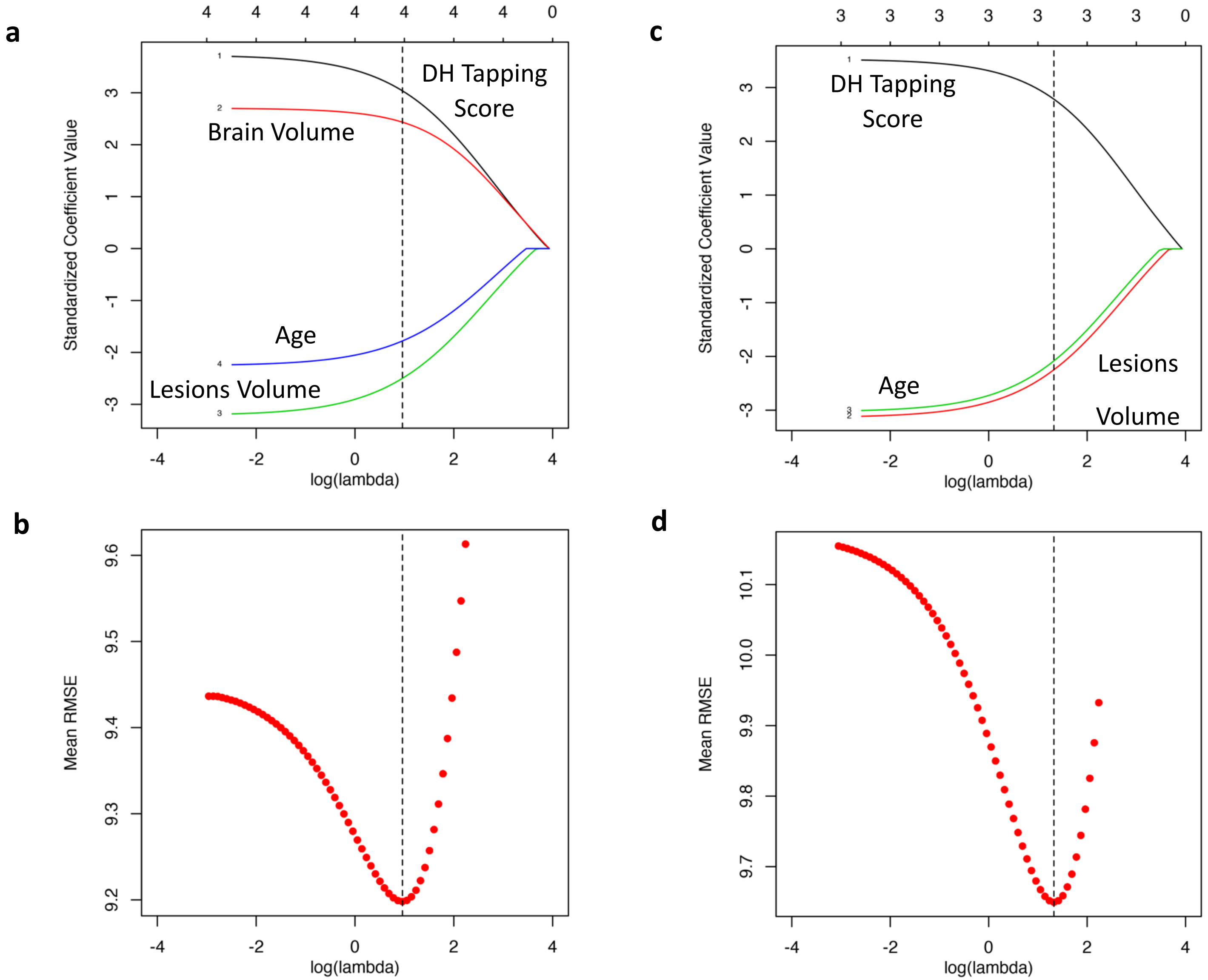

Coefficient path and mean RMSE plots for app SDMT elastic net regressions, with and without tapping score as covariate. **(a,b)** Plots for app SDMT with tapping. **(c,d)** Plots for app SDMT without tapping.

**Mixed-effects model for calculating ICC with sitting number**
app SDMT score = test sitting + (1 | PID)

Supplementary Figure 8

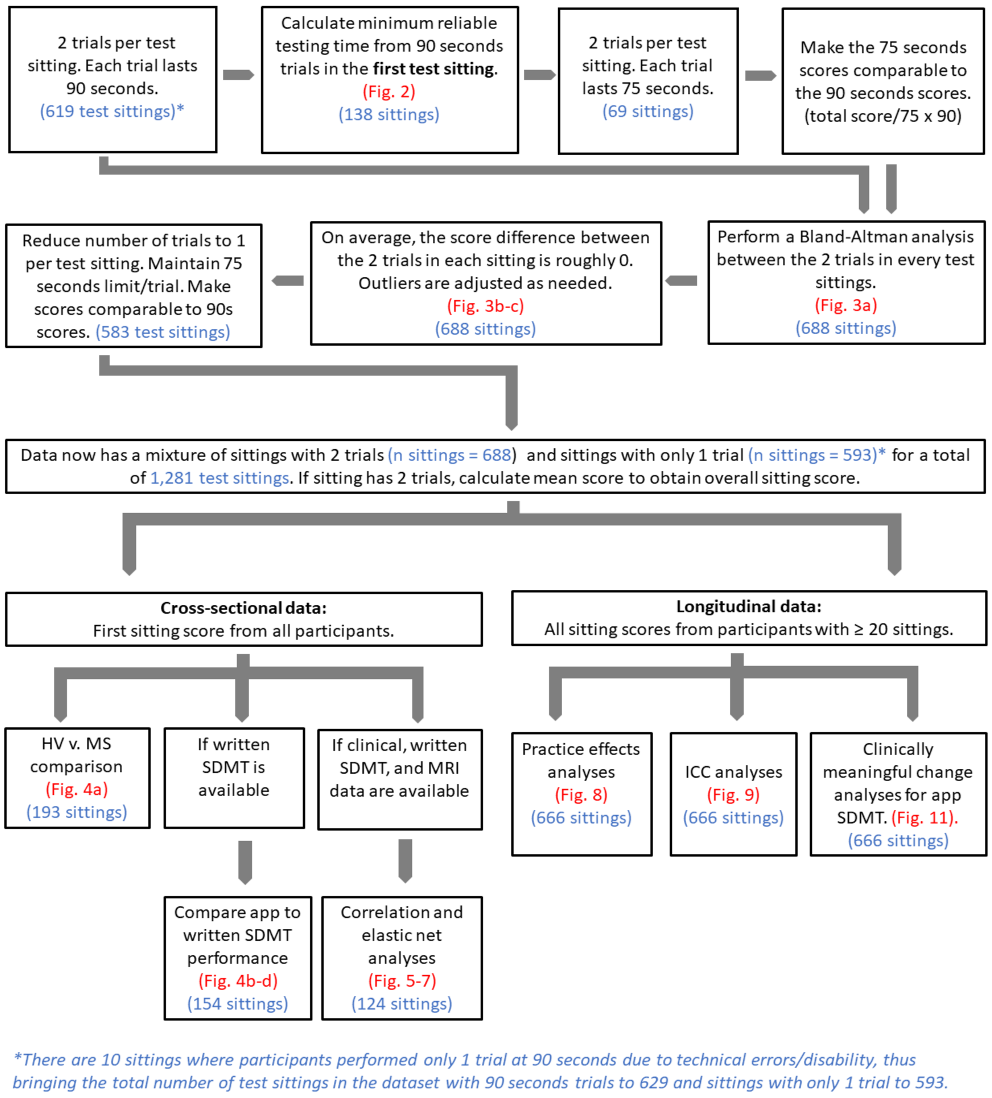

Data Collection and Division Schematic. A time of 75 seconds per trial as well as one trial per setting were found to be optimal. Participants are divided into the cross-sectional and longitudinal cohorts depending on how many sittings they participated in.

Supplementary Figure 9

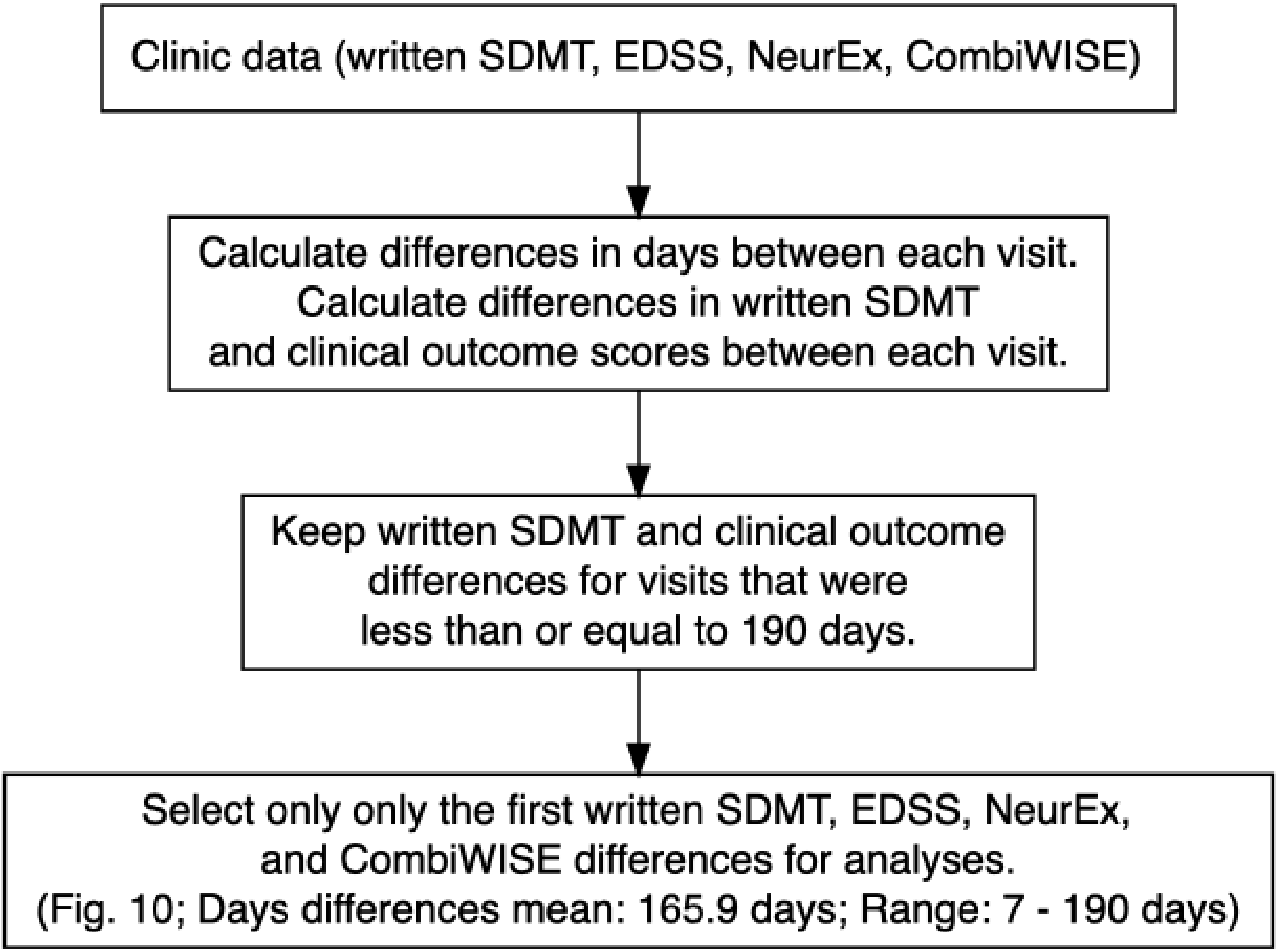

Data cleaning schematic for written SDMT meaningful change analysis

Supplementary Figure 10

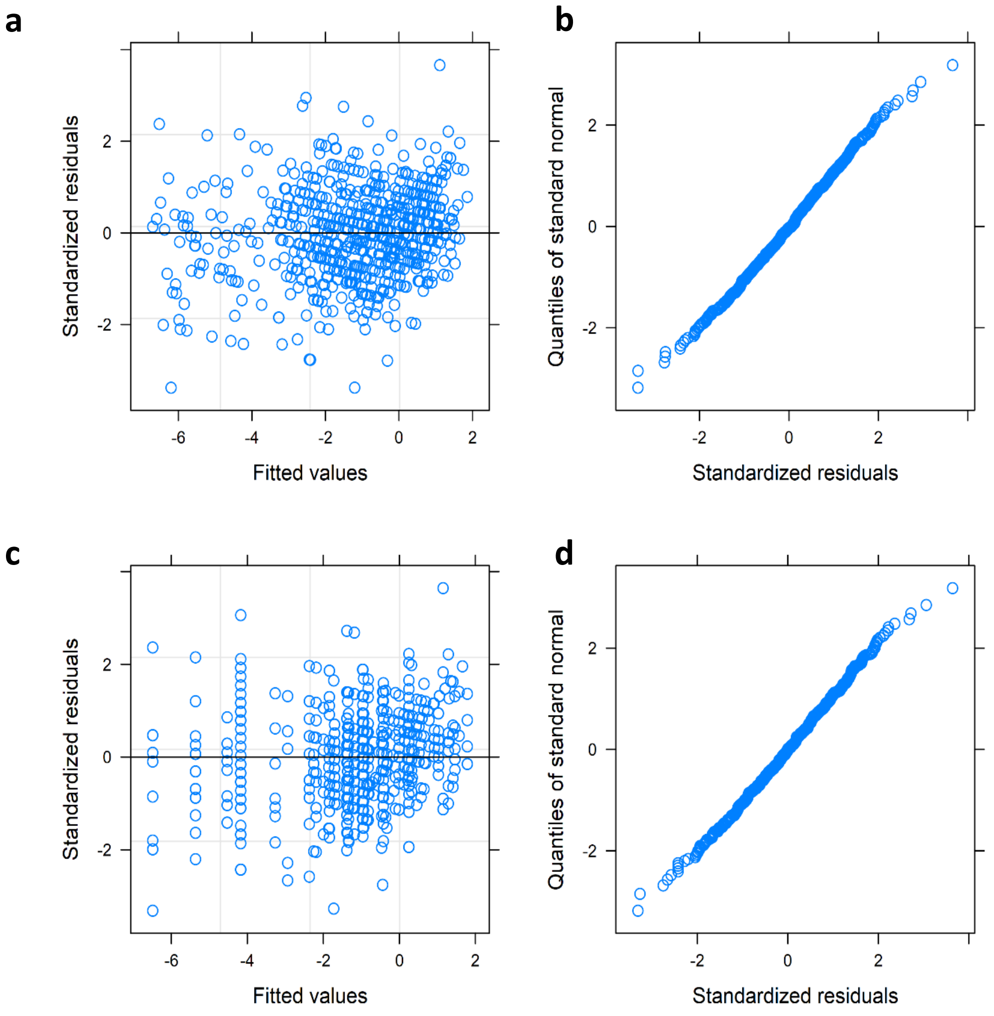

Diagnostic plots for the models that make up the mixed-effects Bland-Altman plot. **(a,b)** Diagnostic plots for the limits of agreement model. **(c,d)** Diagnostic plots for the mean difference model.

Mixed-effects models

For calculating limits of agreement: (trial 2 – trial 1 score) = test sitting + (1 | PID)

For calculating the cohort mean difference: (trial 2 – trial 1 score) = 1 + (1 | PID)

Supplementary Figure 11

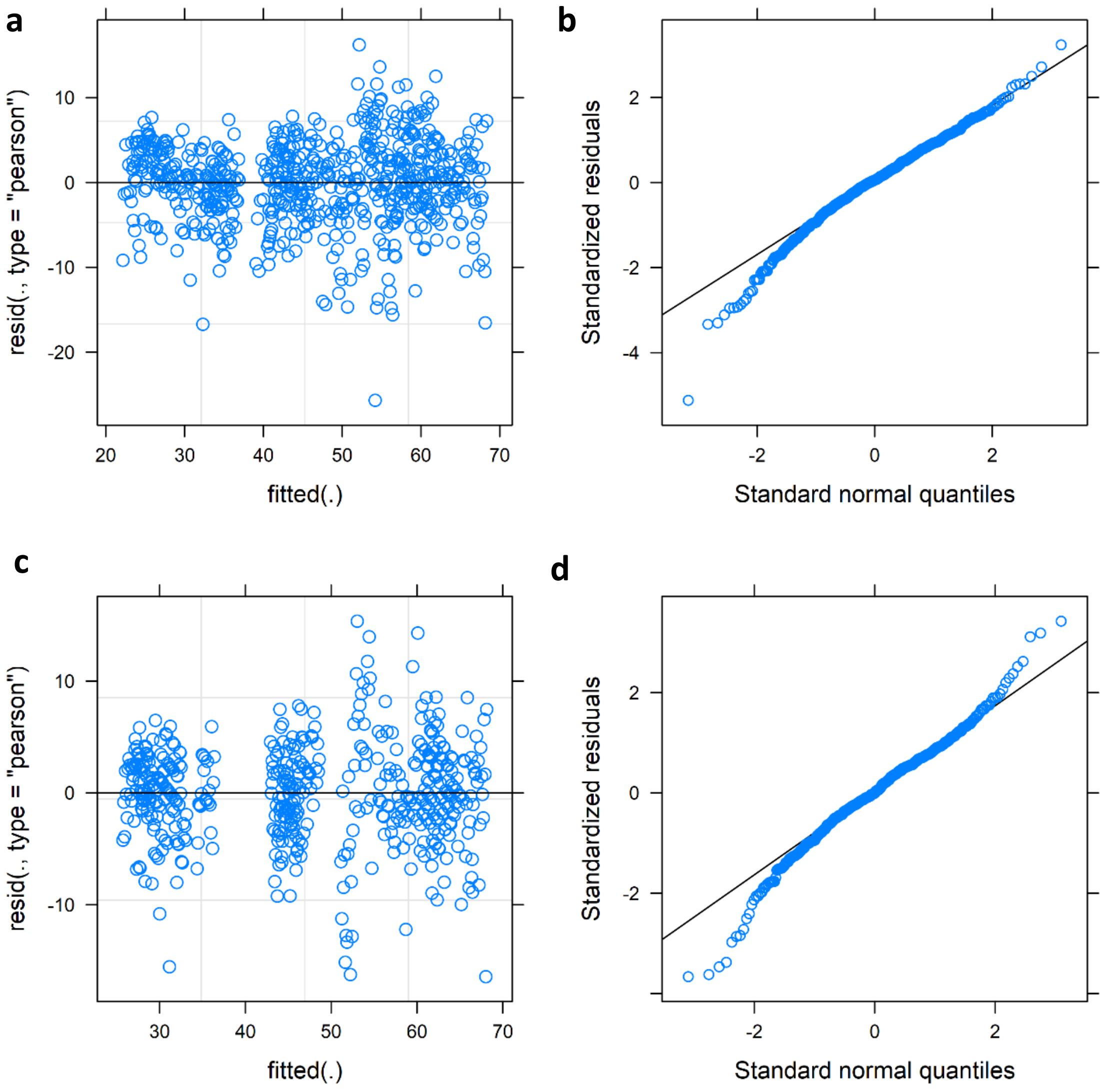

Diagnostic plots for the mixed-effects model that calculate ICC based on scores across number of sittings. **(a,b)** Diagnostic plots for model that includes all longitudinal data. **(c,d)** Diagnostic plots for model that includes only post-learning data.

Supplementary Figure 12

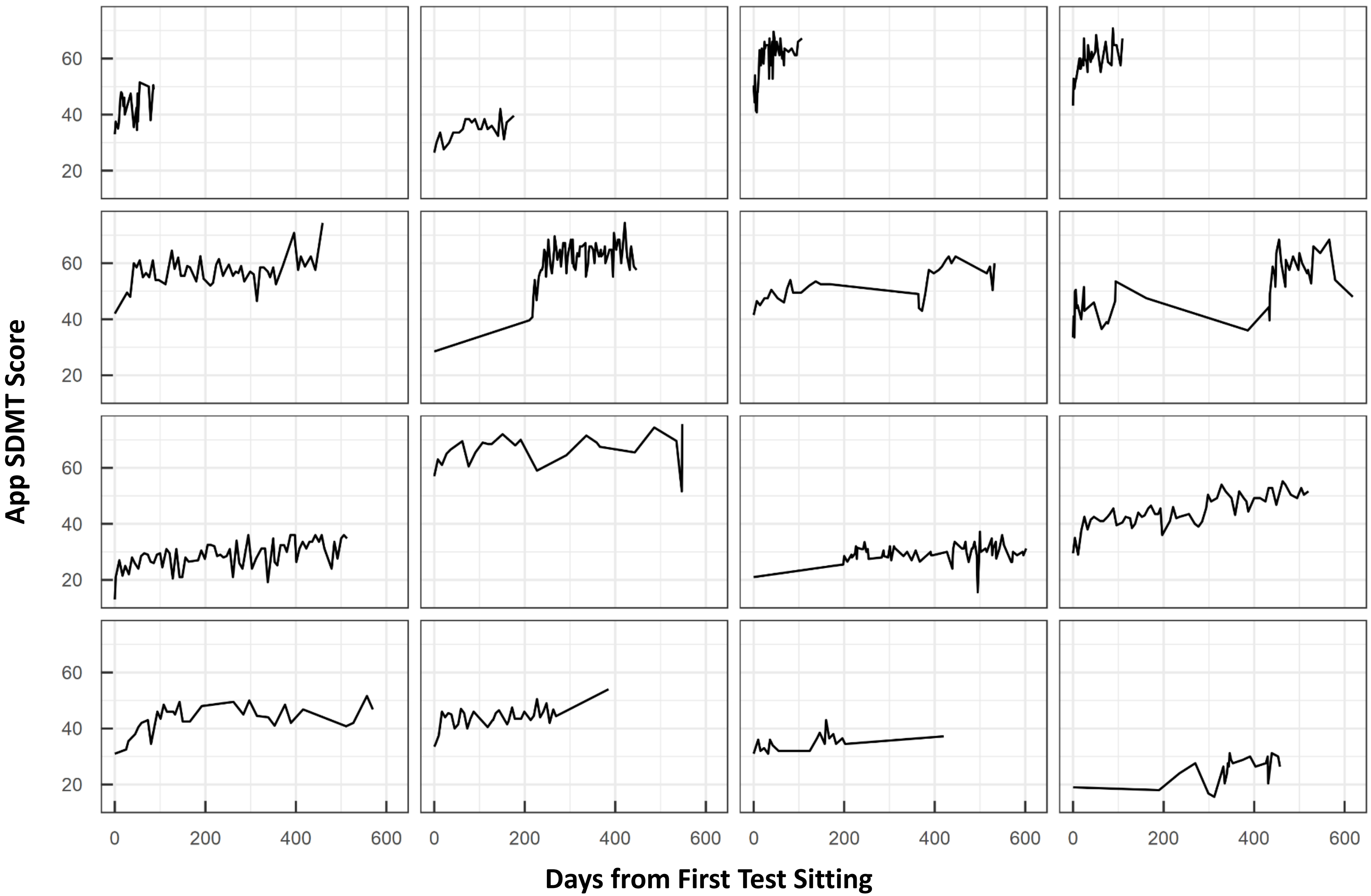

Longitudinal app SDMT scores plotted against days from first test sitting of sitting number. Each panel corresponds with a patient.

**Mixed-effects model for calculating ICC days difference**
app SDMT score = days since first sitting + (1 | PID)

Supplementary Figure 13

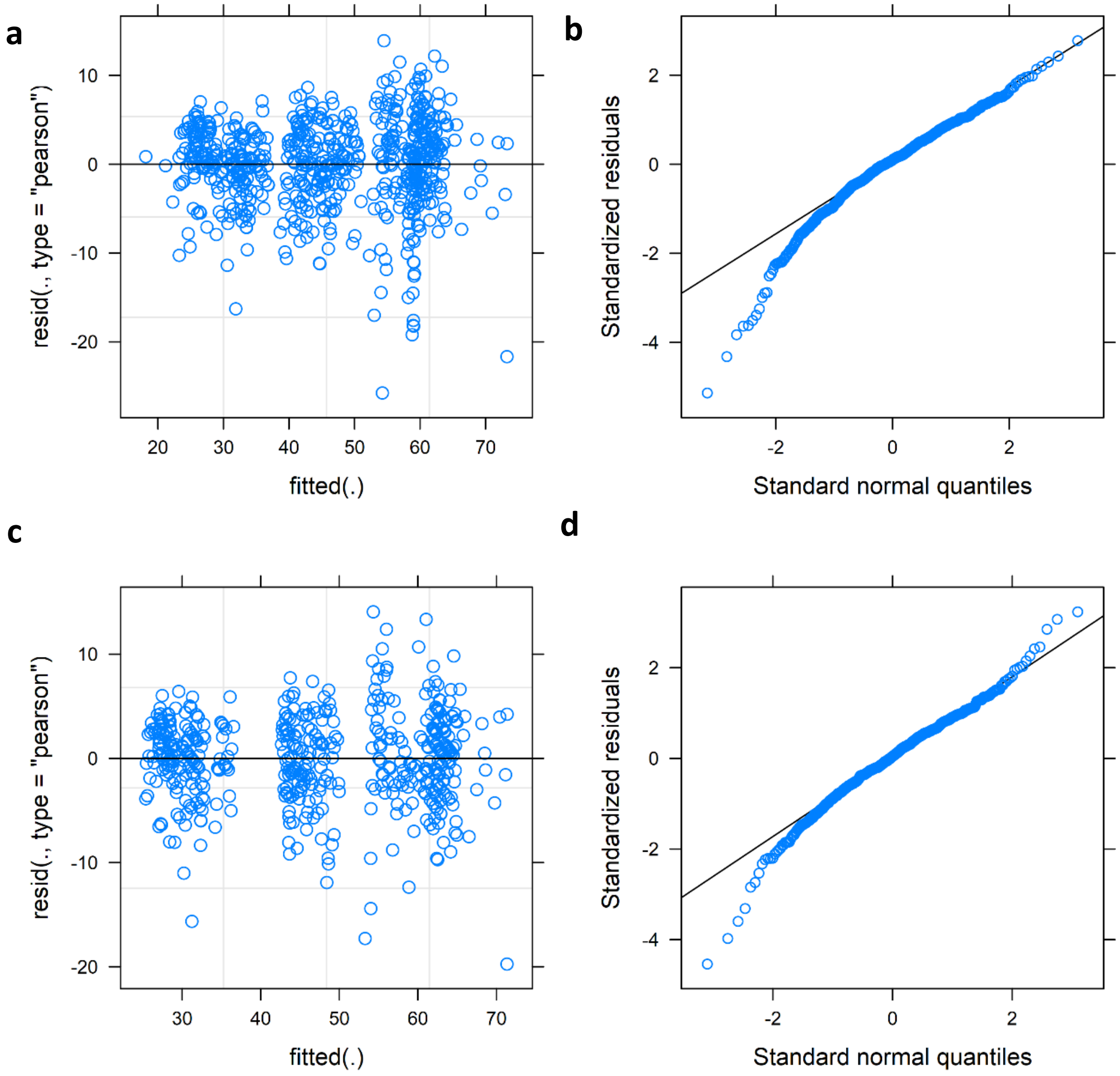

Diagnostic plots for the mixed-effects model that calculate ICC based on scores across different days. **(a,b)** Diagnostic plots for model that includes all longitudinal data. **(c,d)** Diagnostic plots for model that includes only post-learning data (determined using the model in suppl. 9). ICC with all data: 0.88. ICC post-learning: 0.91.

Supplementary Figure 14

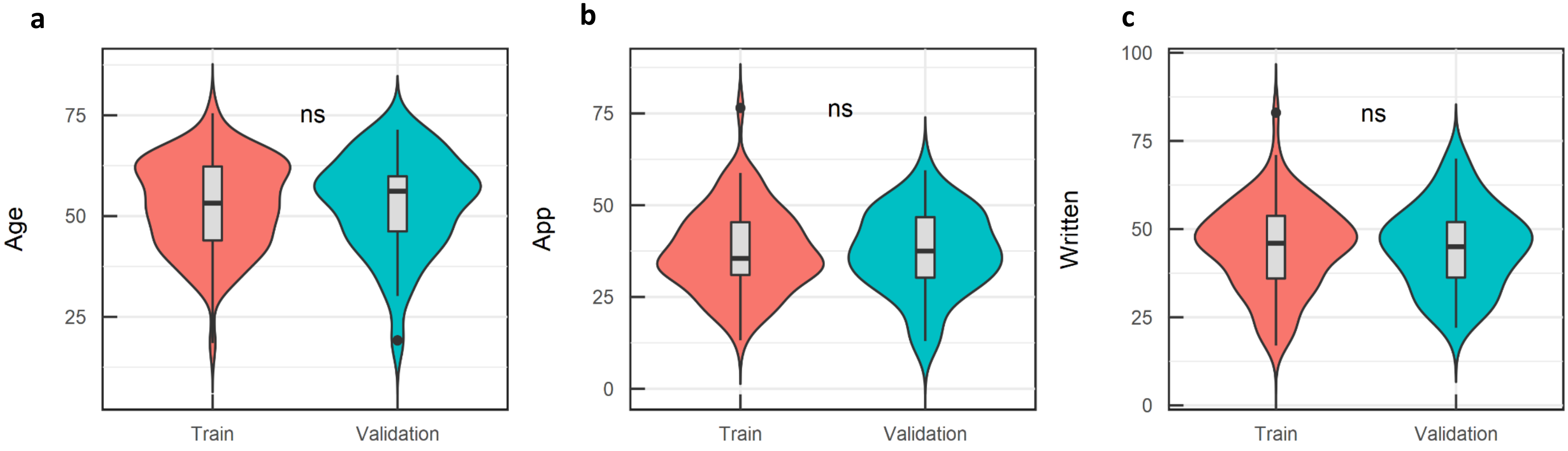

Age and SDMT distributions (app and written) between the cohorts in elastic net regression. The training cohort has 86 individuals, and the validation set has 38 individuals. Wilcoxon rank-sum test determined that age and SDMT scores were not significantly different (ns) between these groups.
