# Supplementary material for: Smartphone-based symbol-digit modalities test reliably captures brain damage in multiple sclerosis
Source: NPJ Digit Med. 2021 Feb 24;4:36. doi: 10.1038/s41746-021-00401-y (PMC7904910; doi:10.1038/s41746-021-00401-y)
Supplement: Supplementary file 2 — Reporting Summary [file 41746_2021_401_MOESM2_ESM.pdf]

nature research

Corresponding author(s): Bibi Bielekova, MD

Last updated by author(s): Dec 29, 2020

Reporting Summary

Nature Research wishes to improve the reproducibility of the work that we publish. This form provides structure for consistency and transparency in reporting. For further information on Nature Research policies, see our Editorial Policies and the Editorial Policy Checklist.

Statistics

For all statistical analyses, confirm that the following items are present in the figure legend, table legend, main text, or Methods section.

n/a	Confirmed	
☐	☒	The exact sample size (*n*) for each experimental group/condition, given as a discrete number and unit of measurement
☐	☒	A statement on whether measurements were taken from distinct samples or whether the same sample was measured repeatedly
☐	☒	The statistical test(s) used AND whether they are one- or two-sided<br>*Only common tests should be described solely by name; describe more complex techniques in the Methods section.*
☐	☒	A description of all covariates tested
☐	☒	A description of any assumptions or corrections, such as tests of normality and adjustment for multiple comparisons
☐	☒	A full description of the statistical parameters including central tendency (e.g. means) or other basic estimates (e.g. regression coefficient) AND variation (e.g. standard deviation) or associated estimates of uncertainty (e.g. confidence intervals)
☐	☒	For null hypothesis testing, the test statistic (e.g. *F*, *t*, *r*) with confidence intervals, effect sizes, degrees of freedom and *P* value noted<br>*Give P values as exact values whenever suitable.*
☒	☐	For Bayesian analysis, information on the choice of priors and Markov chain Monte Carlo settings
☒	☐	For hierarchical and complex designs, identification of the appropriate level for tests and full reporting of outcomes
☐	☒	Estimates of effect sizes (e.g. Cohen's *d*, Pearson's *r*), indicating how they were calculated

Our web collection on statistics for biologists contains articles on many of the points above.

Software and code

Policy information about availability of computer code

Data collection	All data was collected via a smartphone app, developed in-house called NeuFun. The app was written in Kotlin and Java, using the latest Android Studio integrated development environment. The app is compatible with Android 9 and up. App testing was executed on Google Pixel XL/2XL. The software is available for non-commercial collaborators under NIAID technology transfer policy.
Data analysis	R Development Core Team, R: A Language and Environment for Statistical Computing. 2018. Code available (deposited to GitHub repository)

For manuscripts utilizing custom algorithms or software that are central to the research but not yet described in published literature, software must be made available to editors and reviewers. We strongly encourage code deposition in a community repository (e.g. GitHub). See the Nature Research guidelines for submitting code & software for further information.

Data

Policy information about availability of data

All manuscripts must include a data availability statement. This statement should provide the following information, where applicable:

- Accession codes, unique identifiers, or web links for publicly available datasets
- A list of figures that have associated raw data
- A description of any restrictions on data availability

Data used for all analyses can be found in the Supplementary Files. The app is available to all interested research collaborators under NIAID non-commercial limited use agreement. Please reach out to the corresponding author. Raw Data available uploaded to GitHub repository: https://github.com/bielekovaLab/Bielekova-Lab-Code/tree/master/FormerLabMembers/Linh/sdmt_analyses

Field-specific reporting

Please select the one below that is the best fit for your research. If you are not sure, read the appropriate sections before making your selection.

☒ Life sciences ☐ Behavioural & social sciences ☐ Ecological, evolutionary & environmental sciences

For a reference copy of the document with all sections, see nature.com/documents/nr-reporting-summary-flat.pdf

Life sciences study design

All studies must disclose on these points even when the disclosure is negative.

Sample size	There was not a pre-determination of sample size, but the statistical-learning models were validated in the independent cohort.
Data exclusions	All subjects/all available data were included in the analyses
Replication	The Elastic net (EN)-based models were replicated in the independent validation cohort.
Randomization	*Describe how samples/organisms/participants were allocated into experimental groups. If allocation was not random, describe how covariates were controlled OR if this is not relevant to your study, explain why.*
Blinding	All subjects were assigned alphanumeric code for generation of smartphone and MRI data. Clinicians were blinded to the smartphone and MRI data. Smartphone test administrators were blinded to clinical and MRI data. Investigators who generated volumetric MRI data were blinded both to clinical and smartphone data.

Reporting for specific materials, systems and methods

We require information from authors about some types of materials, experimental systems and methods used in many studies. Here, indicate whether each material, system or method listed is relevant to your study. If you are not sure if a list item applies to your research, read the appropriate section before selecting a response.

Materials & experimental systems

n/a	Involved in the study	
☒	☐	Antibodies
☒	☐	Eukaryotic cell lines
☒	☐	Palaeontology and archaeology
☒	☐	Animals and other organisms
☐	☒	Human research participants
☐	☒	Clinical data
☒	☐	Dual use research of concern

Methods

n/a	Involved in the study	
☒	☐	ChIP-seq
☒	☐	Flow cytometry
☐	☒	MRI-based neuroimaging

Human research participants

Policy information about studies involving human research participants

Population characteristics	Please refer to table 2 in our manuscript for details about demographics.
Recruitment	All participants were recruited via the protocol NCT00794352 listed on ClinicalTrials.gov.
Ethics oversight	Central Institutional Review Board of the National Institutes of Health (NIH)

Note that full information on the approval of the study protocol must also be provided in the manuscript.

Clinical data

Policy information about clinical studies

All manuscripts should comply with the ICMJE guidelines for publication of clinical research and a completed CONSORT checklist must be included with all submissions.

Clinical trial registration	NCT00794352
Study protocol	https://www.clinicaltrials.gov/ct2/show/NCT00794352
Data collection	This is an ongoing study. Data collection for the phone app was done at the NIH in Bethesda, Maryland. Phone app data collection began in late 2017.

Outcomes	The clinical, SDMT and MRI outcomes were pre-defined in the protocol. The SDMT psychometric analyses were based on the published review of Multiple Sclerosis Outcome Assessment Committee we cite in the paper.

Magnetic resonance imaging

Experimental design

Design type	Prospective acquisition of volumetric brain MRI data
Design specifications	Not applicable (this was structural imaging, not functional MRI)
Behavioral performance measures	None (this was structural imaging, not functional MRI)

Acquisition

Imaging type(s)	Structural, volumetric
Field strength	3T
Sequence & imaging parameters	T1 magnetization-prepared rapid gradient-echo (MPRAGE), or fast spoiled gradient-echo (FSPGR) images and T2 weighted three-dimensional fluid attenuation inversion recovery (3D FLAIR)
Area of acquisition	Whole brain

Diffusion MRI ☐ Used ☒ Not used

Preprocessing

Preprocessing software	Locally anonymized and encrypted DICOM files were analyzed by an automated segmentation algorithm LesionTOADS implemented into a cloud service for medical image processing by QMENTA (www.qmenta.com).
Normalization	NA
Normalization template	NA
Noise and artifact removal	NA
Volume censoring	NA

Statistical modeling & inference

Model type and settings	NA - MRI results were used as outcome in EN models
Effect(s) tested	*Define precise effect in terms of the task or stimulus conditions instead of psychological concepts and indicate whether ANOVA or factorial designs were used.*

Specify type of analysis: ☒ Whole brain ☐ ROI-based ☐ Both

Statistic type for inference (See Eklund et al. 2016)	*Specify voxel-wise or cluster-wise and report all relevant parameters for cluster-wise methods.*
Correction	*Describe the type of correction and how it is obtained for multiple comparisons (e.g. FWE, FDR, permutation or Monte Carlo).*

Models & analysis

n/a	Involved in the study
☒	☐ Functional and/or effective connectivity
☒	☐ Graph analysis
☒	☐ Multivariate modeling or predictive analysis
